# Proton-irradiated breast cells: molecular points of view

**DOI:** 10.1093/jrr/rrz032

**Published:** 2019-05-28

**Authors:** Valentina Bravatà, Francesco P Cammarata, Luigi Minafra, Pietro Pisciotta, Concetta Scazzone, Lorenzo Manti, Gaetano Savoca, Giada Petringa, Giuseppe A P Cirrone, Giacomo Cuttone, Maria C Gilardi, Giusi I Forte, Giorgio Russo

**Affiliations:** 1 Istituto di Bioimmagini e Fisiologia Molecolare-Consiglio Nazionale delle Ricerche (IBFM-CNR), Cefalù (PA), Italy; 2 National Institute for Nuclear Physics, Laboratori Nazionali del Sud, INFN-LNS, Catania, Italy; 3 Department of Biopathology and Medical Biotechnology, Palermo University, Palermo, Italy; 4 Department of Physics, University of Naples Federico II, via Cintia, Naples, Italy; 5 Nuclear Medicine, San Raffaele Scientific Institute, Milan, Italy

**Keywords:** breast cancer, radiation, cDNA microarray, gene signature, proton therapy

## Abstract

Breast cancer (BC) is the most common cancer in women, highly heterogeneous at both the clinical and molecular level. Radiation therapy (RT) represents an efficient modality to treat localized tumor in BC care, although the choice of a unique treatment plan for all BC patients, including RT, may not be the best option. Technological advances in RT are evolving with the use of charged particle beams (i.e. protons) which, due to a more localized delivery of the radiation dose, reduce the dose administered to the heart compared with conventional RT. However, few data regarding proton-induced molecular changes are currently available. The aim of this study was to investigate and describe the production of immunological molecules and gene expression profiles induced by proton irradiation. We performed Luminex assay and cDNA microarray analyses to study the biological processes activated following irradiation with proton beams, both in the non-tumorigenic MCF10A cell line and in two tumorigenic BC cell lines, MCF7 and MDA-MB-231. The immunological signatures were dose dependent in MCF10A and MCF7 cell lines, whereas MDA-MB-231 cells show a strong pro-inflammatory profile regardless of the dose delivered. Clonogenic assay revealed different surviving fractions according to the breast cell lines analyzed. We found the involvement of genes related to cell response to proton irradiation and reported specific cell line- and dose-dependent gene signatures, able to drive cell fate after radiation exposure. Our data could represent a useful tool to better understand the molecular mechanisms elicited by proton irradiation and to predict treatment outcome

## INTRODUCTION

The idea that genes may function as biomarkers of disease response provides the rationale for the development of molecularly based signatures to predict response to radiation treatment in cancer, including breast cancer (BC). Moreover, microarray-based expression studies have demonstrated their relevance in the comprehension of cancer behavior as well as in cancer care. It is well known that BC is the most common cancer in women, highly heterogeneous at both the clinical and molecular level and showing distinct subtypes associated with different clinical outcomes; hence the need to develop targeted therapeutic strategies [1, 2]. Gene expression profile (GEP) studies allowed BC patients to be divided into clinically relevant subtypes because of their distinct gene expression patterns, associated with different prognoses. In turn, successful applications of specific molecularly based signatures in BC care were reported by the development of specific assays, such as OncotypeDx, MammaPrint and Prosigna, which have revolutionized the decision-making process on the necessity of an adjuvant chemotherapy in BC patients [3].

Ionizing radiation (IR) delivered during radiation therapy (RT) plays a key role in the therapeutic treatments for many types of cancer, including BC. Although technological advances in radiation delivery have decisively enhanced treatment efficacy, the current scenario still presents standard RT schedules that do not take into account the specific molecular subtypes of patients with cancer in the same anatomic position (such as breast, prostate, etc.) [4]. However, it is now well known that tumor heterogeneity, in terms of both clinical and molecular characteristics, strongly affects treatment outcome. Indeed, tumor radiosensitivity also depends on many factors linked to biological characteristics. For example, in BC, cell fate after radiation exposure depends on many factors such as hormone receptor status (estrogen and progesterone receptors, and human epidermal growth factor receptor 2), the number of tumor cancer stem cells present before initiation of RT and their ability to repopulate during the course of RT, effects of the tumor microenvironment such as hypoxia, stromal interaction and variations in the intrinsic sensitivity of cells to radiation, modulation of DNA repair or other cell survival pathways [5–8].

Thus, the choice of a unique RT plan, common to all BC patients, may not be the best option. In addition, the advent of hadrontherapy, particularly with the use of accelerated proton beams, has led to a number of potential advantages over conventional (photon/electron-based) RT for cancer, the most important of which is arguably a more localized delivery of the radiation dose with the consequent sparing of healthy tissues and/or organs at risk [9–13]. In BC treatment, the prospective use of proton therapy in place of conventional RT would result in a lower radiation dose to the heart and lungs, especially if the tumor is located in the left breast [14, 15]. Because of such advantages and due to clinically encouraging results, proton therapy is currently used for several cancers and its use is rapidly growing [https://www.ptcog.ch/index.php/facilities-in-operation, accessed June 2018].

However, few data are available regarding proton-induced molecular changes, particularly in breast cells, a topic that therefore deserves to be accurately described.

In addition, based on our previous findings, IR exposures could stimulate the secretion of numerous inflammatory factors, which can affect cell fate via multiple pathways and may thus influence tumor progression control and the overall therapy outcome [16–20]. These secreted factors may also interact with surrounding cells and, hence, may damage unirradiated tissue via the bystander effect [17]. To our knowledge, the immunological response of BC after proton irradiation has yet to be investigated. Thus, the identification of specific proton irradiation prognostic biomarkers and gene signatures, predictive of RT efficacy, is of great usefulness in future clinical practice. Equally important, the choice of an appropriate RT treatment plan, based also on the knowledge of biological features of the tumors to be treated, is necessary in order to increase the chances of success [3].

In this scenario, the aim of this study was to describe the dose–response effects on cell survival induced by proton beam irradiation and, for the first time to our knowledge, the radiation-induced GEPs and immunological molecules profiles produced by the MCF10A mammary non-tumorigenic cell line, and MCF7 (not metastatic, luminal, ER+/PR+/HER2−) and MDA-MB-231 (metastatic, basal, triple negative) BC cell lines with different aggressive phenotypes [21], after graded doses of proton irradiation. In particular, for the molecular investigations, three radiation doses were selected based on the following criteria: 0.5 Gy to evaluate the effects of low doses; 2 Gy as it represents the fractionated dose in RT of breast cancer; 9 Gy to evaluate the effects of high doses also to be compared with our previous studies on electron beams. [17, 22, 23]. Our results highlighted the global molecular and immunological response of tumorigenic and non-tumorigenic BC cell lines to proton therapy.

## MATERIALS AND METHODS

### Proton irradiation set-up

Proton irradiations were performed using the 62 MeV proton beam generated by the superconducting cyclotron clinically used at the CATANA (Centro di AdroTerapia ed Applicazioni Nucleari Avanzate) eye proton therapy facility of the Italian Institute for Nuclear Physics in Catania, Italy [20–22]. The protons were accelerated by a superconductive cyclotron; the beam was converted into a uniform clinical beam, according to the international guidelines, able to cover the entire target region passing through different passive elements [24–26]. Flasks were irradiated in the upright position facing the collimated beam exit by delivering separate shots to cover the flask surface entirely; an ad-hoc remotely controlled positioning system ensured that after each shot, the flask was moved so that the next shot would hit the adjacent area. Uniformity in dose distribution and inter-shot reproducibility was routinely checked prior to each experimental run using radiochromic films. Dosimetry was also performed: the lateral beam profile was verified using a semiconductor diode, while the depth dose profiles and dosimetric calibrations were performed using a motorized Markus chamber within a water tank. The dosimetric system was calibrated under reference conditions defined by the International Atomic Energy Agency Technical Reports Series No. 398 ‘Absorbed Dose Determination in External Beam Radiotherapy’ [27]. Cell irradiations were conducted placing the cells at the middle of the spread-out Bragg peak (SOBP, 1 cm width), to simulate a clinical condition, with dose values of 0.5, 2, 4, 6 and 9 Gy, and a dose rate of 15 Gy min^–1^.

### Cell culture and clonogenic survival assay

**Fig. 1.**
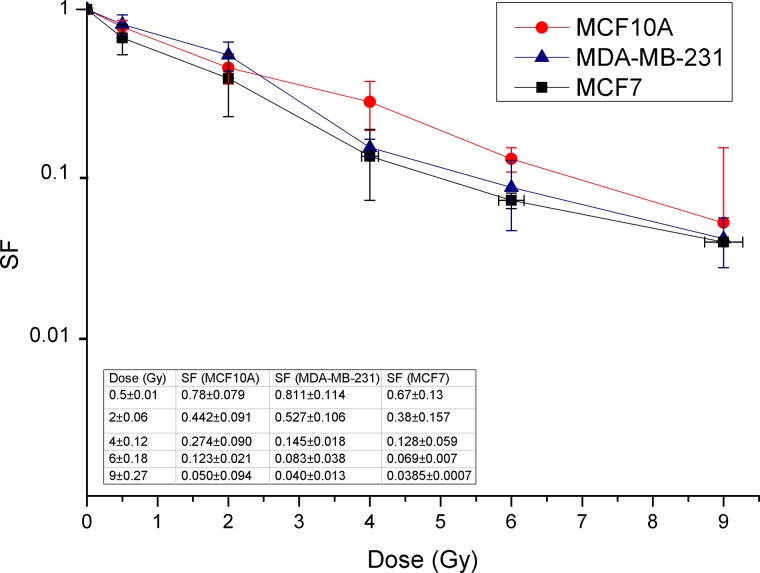
Dose-dependent survival curves of MCF10A, MCF7 and MDA-MB-231 cells after proton irradiation with doses of 0.5, 2, 4, 6 and 9 Gy at the therapeutic middle SOBP.

The human non-tumorigenic breast epithelial MCF10A cell line and the human breast adenocarcinoma MCF7 and MDA-MB-231 cell lines were purchased from the American Type Culture Collection (ATCC) and cultured according to the manufacturer’s instructions (ATCC, Manassas, VA, USA) as previously reported [13]. Cells were maintained in an exponentially growing state at 37°C in a 5% CO_2_ incubator; 2 days before irradiation they were seeded in T25 tissue culture flasks at 3–5×10^5^ cells per flask. Cell survival was evaluated by clonogenic assay performed as previously described [22, 23, 28]. Briefly, 24 h after irradiation, cells were detached, counted by a hemocytometer and re-plated in triplicate at opportune densities according to the dose delivered (200–2000 cells per well) in a 6-well plate to assay the surviving fraction (SF). Untreated cells (basal) were used as control in order to evaluate the plating efficiency (PE). Cells were allowed to form colonies under normal cell culture conditions for 10–12 days and then were fixed and stained for 30 min with 6% glutaraldehyde and 0.5% crystal violet (both from Sigma-Aldrich, St. Louis, MO, USA). Colonies with >50 cells were counted manually under a Zeiss Axiovert phase-contrast microscope (Carl Zeiss, Göttingen, Germany). Dose–response data shown in the graph of Fig. 1 are generated from the mean of three independent experiments. The error bars for each cell sample are on the *x*-axis for the dosimetric error and on the *y*-axis for the error of the SFs calculated with the error propagation method.

### Cytokine, chemokine and growth factor analysis by Luminex

Cells were collected at 24, 48 and 72 h post-irradiation. Twenty-four hours before irradiation, the growth medium was replaced with fresh medium. At the above times after exposure, irradiated conditioned medium (ICMs) was collected. For each cell line, one flask with the same cell number compared with treated samples was seeded and grown under the same experimental conditions. Thus, their complete conditioned medium (CM) was collected and used as control (basal, i.e. untreated). Complete media without cells were incubated under the same experimental conditions and used as the blank controls. CM and ICM were stored at −80°C until use. Immediately before the cytokine assay, thawed samples were centrifuged at 12 000 rpm for 5–10 min to allow precipitation of any lipid excess and tested for the following panel of 17 cytokines, chemokines and growth factors: interleukin (IL)-1β, IL-2, IL-4, IL-5, IL-6, IL-7, IL-8, IL-10, IL-12(p70), IL-13, IL-17, interferon (IFN)-γ, tumor necrosis factor (TNF)-α, monocyte chemoattractant protein-1 (MCP-1), macrophage inflammatory protein-1β (MIP-1β), granulocyte–macrophage colony-stimulating factor (GM-CSF) and granulocyte colony-stimulating factor (G-CSF). The assay was carried out using the Luminex system (BioRad, Munchen, Germany) according to the manufacturer’s instructions, as previously described [17]. The assay was performed using an eight-point standard curve for every molecule. Data were evaluated using the Bio-Plex Manager software (BioRad). Standards, internal controls and samples are calculated as means of duplicate measurements. Data reported in Tables 1, 2 and 3 for each time point correspond to normalized values of expression levels. In particular, the means of duplicates for each cytokine/time point of treated samples have been normalized with respect to the means of duplicates for each cytokine of untreated controls collected on the day of irradiation (time point 0). Furthermore, Supplementary file 2 contains a second-order polynomial fitting analysis conducted for each immunological molecule, in order to study their trend according to time and dose delivered.

**Table 1.** MCF10A cytokine, chemokine and growth factor profiles induced by proton treatments

	MCF10A non-tumorigenic mammary epithelial cells								
	24 h			48 h			72 h		
Analytes	0.5 Gy	2 Gy	9 Gy	0.5 Gy	2 Gy	9 Gy	0.5 Gy	2 Gy	9 Gy
**IL-1β**	0.78	0.69	0.81	0.94	0.95	1.34	1.08	1.17	1.69
**IL-6**	0.91	0.83	0.86	0.82	0.98	2.48	0.78	0.97	2.89
**TNF-α**	0.78	0.65	0.83	0.96	0.94	1.32	1.08	1.21	1.53
**IL-12**	0.29	0.11	0.47	0.64	0.29	1.00	0.64	0.64	1.18
**IFN-γ**	0.82	0.71	0.82	0.95	0.94	1.23	1.07	1.17	1.39
**IL-4**	0.80	0.74	0.84	0.97	0.96	1.27	1.09	1.19	1.49
**IL-10**	0.43	0.32	0.54	0.43	0.49	1.24	0.60	0.94	1.18
**IL-13**	0.77	1.00	0.77	0.77	0.77	1.00	0.77	1.00	1.23
**IL-8**	0.99	0.87	1.06	1.17	1.28	2.15	1.37	1.65	3.69
**MCP-1**	0.77	0.65	0.67	1.31	1.11	1.12	3.53	4.56	3.11
**MIP-1β**	0.18	0.00	0.33	0.84	0.56	2.01	1.30	1.64	2.82
**IL-2**	0.72	0.60	0.75	0.90	0.87	1.23	0.99	1.11	1.38
**IL-7**	0.66	0.48	0.66	1.00	0.77	1.16	1.32	1.32	1.27
**IL-17**	0.68	0.56	0.78	1.04	0.96	1.70	1.27	1.47	2.20
**G-CSF**	0.70	0.57	0.79	0.99	0.90	1.91	1.22	1.61	3.77
**GM-CSF**	0.90	0.86	0.86	1.02	1.02	1.29	1.10	1.10	1.39

IL-5 was undetectable.

Values (in term of pg ml^–1^) were normalized using CM of untreated MCF10A cells.

**Table 2.** MCF7 cytokine, chemokine and growth factor profile induced by proton treatments

	MCF7 breast cancer cell line								
	24 h			48 h			72 h		
Analytes	0.5 Gy	2 Gy	9 Gy	0.5 Gy	2 Gy	9 Gy	0.5 Gy	2 Gy	9 Gy
**IL-6**	1.18	1.29	1.25	1.67	1.64	3.33	3.62	4.31	7.36
**TNF-α**	0.38	0.69	1.00	0.69	0.84	0.69	0.69	1.00	1.33
**IFN-γ**	0	0	0	0	0.17	1.00	0	1.00	0
**IL-8**	0.77	0.59	1.45	0.91	1.23	1.45	2.00	2.55	3.50
**MCP-1**	0.00	0.00	0.11	0.40	1.00	1.00	3.26	2.56	0.00
**IL-7**	0.00	0.09	0.09	0.26	0.26	0.00	0.26	0.09	0.43
**IL-17**	0.80	0.54	0.80	1.07	0.66	0.80	1.34	1.46	0.93
**GM-CSF**	0.00	0.00	0.00	0.08	1.38	1.96	2.77	1.50	0.23

IL-2, IL-4, IL-5, IL-10, IL-12, IL-13, G-CSF, MIP-1b and IL-1β were undetectable.

Values (in term of pg ml^–1^) were normalized using CM of untreated MCF7 cells.

**Table 3.** MDA-MB-231 cytokine, chemokine and growth factor profiles induced by proton treatments

	MDA-MB-231 breast cancer cell line								
	24 h			48 h			72 h		
Analytes	0.5 Gy	2 Gy	9 Gy	0.5 Gy	2 Gy	9 Gy	0.5 Gy	2 Gy	9 Gy
**IL-1β**	1.00	1.28	1.44	5.00	5.28	7.68	7.04	11.84	17.92
**IL-6**	1.25	1.65	1.43	3.94	4.58	4.04	5.48	9.90	8.73
**TNF-α**	1.38	1.57	1.57	3.54	3.74	3.74	4.75	8.49	6.81
**IFN-γ**	0.00	0	0	8.74	10.31	10.31	18.55	40.23	36.28
**IL-4**	1.25	1.14	1.25	1.44	1.78	1.89	1.83	2.67	2.61
**IL-13**	0.13	0.00	0.13	0.00	0.13	0.13	0.13	0.13	0.25
**IL-8**	1.12	1.25	2.00	3.38	3.50	5.46	5.05	8.21	10.25
**MCP-1**	1.13	1.19	1.63	1.63	1.91	2.09	2.09	3.15	3.21
**IL-7**	1.00	1.25	1.63	1.25	1.50	1.56	1.63	2.33	2.21
**G-CSF**	1.11	1.57	1.82	3.65	4.32	5.18	4.99	9.63	9.20
**GM-CSF**	1.04	0.98	1.26	1.14	1.24	1.74	1.33	1.73	2.42

IL-2, IL-5, IL-10, IL-12, IL-17 and MIP-1b were undetectable.

Values (in term of pg ml^–1^) were nomralized using CM of untreated MDA-MB-231 cells.

### Whole-genome cDNA microarray expression analysis

To study molecular pathways and cell networks activated at the transcriptional level following proton beam irradiations, gene expression experiments by cDNA microarray were conducted in order to select potential new biomarkers of radiosensitivity and radioresistance as previously described [22, 23]. Twenty-four hours after irradiation with 0.5, 2 and 9 Gy, MCF10A, MCF7 and MDA-MB-231 cell lines were harvested, counted and the pellet stored immediately at −80°C. Total RNA was extracted from cells using Trizol and the RNeasy mini kit according to the manufacturer’s guidelines (Invitrogen). RNA concentration and purity were determined spectrophotometrically using a Nanodrop ND-1000 (Thermo Scientific Open Biosystems, Lafayette, CO, USA), and RNA integrity, measured as RNA integrity number (RIN) values, was assessed using a Bioanalyzer 2100 (Agilent Technologies, Santa Clara, CA, USA). Only samples with a maximum RIN of 10 were used for further microarray analysis. A 500 ng aliquot of total RNA was used for conplementary RNA (RNA) synthesis and labeling according to the Agilent Two-Color Microarray-Based Gene Expression Analysis protocol. Samples were labeled with Cy5 dye and control with Cy3 dye (Agilent Technologies). Fluorescent cRNA samples (825 ng) were then hybridized onto Whole Human Genome 4 × 44 K microarray GeneChips (Agilent Technologies) containing all known genes and transcripts of an entire human genome. Six replicates were performed. Array hybridization was conducted for 17 h at 65°C. Images were acquired with a DNA Microarray Scanner with Sure Scan high-Resolution Technology (Agilent Technologies). Statistical data analysis, background correction and normalization of the gene expression profiles (GEPs) were performed using Feature Extraction and GeneSpring software GX 13.0 (Agilent Technologies). Specifically, data were filtered using a two-step procedure: first the entities were filtered based on their flag values P (present) and M (marginal) and then filtered based on their signal intensity values, which enables very low signal values or removal of those that have reached saturation. Statistically significant differences were computed by Student’s *t-*test, and the significance level was set at *P <* 0.05. The false discovery rate (FDR) was used as a multiple test correction method. Genes were identified as being differentially expressed if they showed a fold change (FC) of at least 2 with a *P*-value < 0.05 compared with untreated MCF10A, MCF7 and MDA-MB-231 cell lines used as reference samples. As described above, the same experimental approach was performed for both cell lines used in this project. The data discussed in this publication have been deposited in the National Center for Biotechnology Information Gene Expression Omnibus (GEO) [29] and are accessible through GEO Series accession numbers (GSE116325 and GSE103472). Microarray data are available in compliance with Minimum Information About a Microarray Experiment (MIAME) standards.

### Pathway analyses of GEP lists

Differentially expressed gene lists obtained by GEP analysis were analyzed using Reactome, a tool that provides a comprehensive set of functional annotation for investigators to understand biological meaning behind a large list of genes. Reactome is a free, open-source, open-data, curated and peer-reviewed knowledge base of biomolecular pathways (https://reactome.org/). One of its main priorities is to provide easy and efficient access to its high-quality curated data and to provide intuitive bioinformatics tools for the visualization, interpretation and analysis of pathway knowledge. It also provides a rapid means to reduce large lists of genes into functionally related groups of genes to help unravel the biological content captured by high-throughput technologies such as microarray analyses [30].

### PubMatrix

All genes assayed in this work were analyzed using the PubMatrix tool, as previously described, in order to confirm our assumptions and to study bibliographic relationships between proteins and some selected queries such as IR, radiation, cancer, BC, proton, inflammation, cell cycle and apoptosis [31].

## RESULTS

### Cell survival

In order to test the effects of the proton beam irradiation on cell loss of reproductive capacity, MCF10A non-tumorigenic mammary epithelial cells, and MCF7 and MDA-MB-231 tumorigenic BC cell lines were irradiated with the doses of 0.5, 2, 4, 6 and 9 Gy.

Dose–response effects were tested by clonogenic assay, and the SFs obtained revealed varying cellular radiosensitivity according to the breast cell lines analyzed (Fig. 1). In particular, the SFs following exposure to doses of 0.5, 2 and 9 Gy are as follows: MCF10A cells, 0.78 (± 0.08); 0.44 (± 0.09); 0.050 (± 0.09); MCF7 cells, 0.67 (± 0.13); 0.38 (± 0.15); 0.0385 (± 0.0007); and MDA-MB-231 cells, 0.81 (± 0.11); 0.53 (± 0.10); 0.040 (± 0.01), respectively.

### Immunological molecule profiles secreted after radiation treatment

We evaluated the relative expression of cytokines, chemokines and growth factors produced by non-tumorigenic mammary epithelial MCF10A cells and tumorigenic BC MCF7 and MDA-MB-231 cell lines after proton irradiation with the doses of 0.5, 2 and 9 Gy and assayed 24, 48 and 72 h after radiation exposures. The above-mentioned immunological factors were chosen according to their involvement in the cell radiation response, as described by several authors and also by our group [16, 17]. The results of these assays are displayed in Tables 1, 2 and 3. Furthermore, Supplementary file 2 contains a second-order polynomial fitting analysis conducted for each immunological molecule, in order to study their trend according to time and dose delivered.

#### Immunological molecule profiles secreted by the non-tumorigenic breast MCF10A cell line

As shown in Table 1, 16 out of 17 immunological molecules investigated were deregulated in MCF10A cells after proton irradiation, compared with untreated cells. Only IL-5 levels fell below the instrument detection threshold because of a too low secretion in ICM.

In summary, as shown in Table 1 and in Supplementary file 2, a slight increase of cytokine secretion was observed at 48 and 72 h post-irradiation with low doses (0.5 and 2 Gy). However, a rapid increase of all the molecules tested was observable starting from 48 h post-treatment after irradiation with the highest dose used, i.e. 9 Gy, describing a polynomial-type increasing trend of inflammatory molecules (IL-1β, IL-6, TNF-α and IL-17), TH1-type (IL-12 and IFN-γ), TH2-type (IL-4 and IL-10), chemokines (IL-8, MCP-1 and MIP-1β) and growth factors (IL-2, G-CSF and GM-CSF). These findings suggest a time- and dose-dependent secretion of immunological molecules. It is noteworthy that the first temporarily up-regulated molecules, regardless of the dose delivered, are the two chemokines IL-8 and MCP-1, for which the increase is observed as early as 48 h post-treatment.

#### Immunological molecule profiles secreted by the breast cancer MCF7 cell line

As regards the MCF7 BC cell line, Table 2 shows the cytokine, chemokine and growth factor signature for the three doses used (0.5, 2 and 9 Gy), at the chosen post-irradiation time points (24, 48 and 72 h). As displayed, only 8 out of the 17 immunological factors assayed were detectable. In particular, the levels of IL-2, IL-4, IL-5, IL-10, IL-12, IL-13, G-CSF, MIP-1β and IL-1β were undetectable because of their too low secretion in ICM. These results are in line with those recently described by Desai *et al.* regarding the minimal secretion of immunological factors in the ICM by MCF7 cells compared with other human cancer cell lines analyzed after radiation exposure, also described by our group following electron radiation treatments [17–20]. As shown in Table 2 and in Supplementary file 2, polynomial fitting analysis describes an irregular trend for many of the assayed molecules. Only IL-6 and IL-8 seem to be produced in a time- and dose- delivered-dependent manner. In particular, a peak of release was highlighted in ICM for the pro-inflammatory cytokine IL-6 and the chemokines IL-8 and MCP-1 72 h after proton irradiation, as these molecules were up-regulated by a 2-fold factor, compared with CM of untreated MCF7 cells.

#### Immunological molecule profiles secreted by the metastatic breast cancer MDA-MB-231 cell line

As above described, the same Luminex experimental approach was performed for proton-treated MDA-MB-231 BC cells. In detail, Table 3 shows the relative expression of the immunological factors released by cells at 24, 48 and 72 h post-proton irradiation using the doses of 0.5, 2 and 9 Gy. As assayed, 11 out of 17 immunological molecules investigated were deregulated in MDA-MB-231 cells after irradiation, compared with the control. In fact, IL-5, IL-12, IL-10, IL-2, MIP-1β and IL-17 were undetectable, because of their too low secretion in ICM. As also shown in Table 3 and in Supplementary file 2, with the exception of IL-13, all the other factors were up-regulated in a time- and dose increase-dependent manner. Overall, the immune response profile of MDA-MB-231 cells to irradiation was characterized by an earlier activation of almost all the immunological factors found in the ICM; such an increase was evident already 24 h post-treatment, with the exception of IFN-γ and IL-13, becoming consistent especially after 48 and 72 h. These data suggest a time-dependent cytokine signature; however, in the case of MDA-MB-231, the dose effect is less evident, since even for the low doses (0.5 and 2 Gy) there is a conspicuous secretion of the molecules found in the ICM, except for IL-13, with a 3-fold increase for 6 out 12 molecules assayed (IL-1β, IL-6, TNF-α, IFN-γ, IL-8 and G-CSF). Note that the IFN-γ, reached a value of 40.23 for the dose of 2 Gy at the time point of 72 h post-treatment and 36.28 with 9 Gy at the same time point, suggesting the activation of a strong TH1-type response. Overall, increased levels of IL-1β, IL-6, TNF-α, IL-7 and IFN-γ (characterized by a pro-inflammatory behavior), IL-8 and MCP-1 (chemokines) and G-CSF and GM-CSF (growth factors) were observed, especially at 72 h post-treatment at all radiation doses. Hence, MDA-MB-231 cells showed the strongest potentially pro-inflammatory secretion profile compared with the other cell types analyzed. Indeed, these cells produce a large spectrum of inflammatory molecules regardless of the dose delivered, unlike MCF7 and MCF10 for which only IL-6, IL-8 and MCP-1 showed this peculiarity.

### Overview of cDNA microarray gene expression and pathway analysis

#### Non-tumorigenic breast MCF10A cell line

**Fig. 2.**
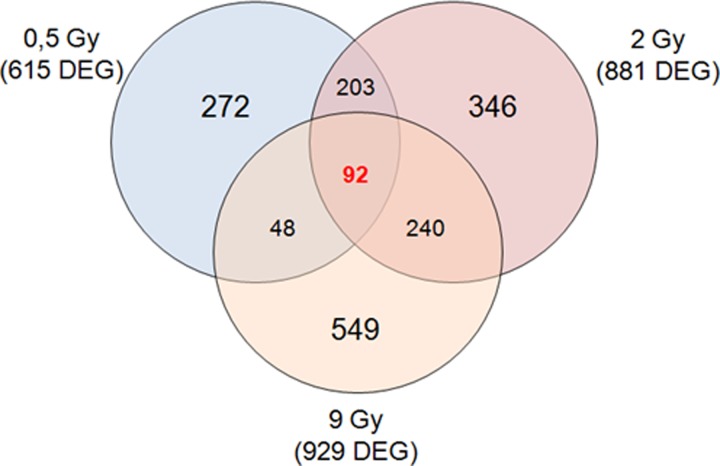
Venn diagram of microarray gene sets of MCF10A cells exposed to 0.5, 2 and 9 Gy of proton irradiation.

In this study, a two-color microarray-based gene expression analysis was conducted on MCF10A cells 24 h post-irradiation with proton beams using 0.5, 2 and 9 Gy of IR doses, compared with untreated MCF10A cells, used as the reference sample. Comparative differential gene expression analysis revealed that multiple genes were significantly altered, by ≥2-fold, compared with untreated cells as follows: MCF10A 0.5 Gy, 615 differentially expressed genes (DEGs; 167 down-regulated and 448 up-regulated); MCF10A 2 Gy, 881 DEGs (224 down-regulated and 657 up-regulated); and MCF10A 9 Gy, 929 DEGs (319 down-regulated and 610-up regulated) (Fig. 2).

Moreover, up- and down-regulated transcripts were selected and grouped according to their involvement in specific biological pathways using the Reactome tool [30] as displayed in Tables 4, 5 and 6. The result of this mapping revealed the involvement of a set of factors controlling cellular processes, such as gene transcription, cell fate, immune response, cell adhesion, migration and cellular trafficking, in comparison with the reference sample. In addition, the GEP lists of MCF10A 0.5 Gy, MCF10A 2 Gy and MCF10A 9 Gy were also analyzed by Venn diagrams in order to identify overlapping deregulated genes among all the configurations assayed in this work, as shown in Fig. 2. Summarizing, in MCF10A cells, some common genes were deregulated by proton irradiation between 0.5 and 2 Gy (203 common genes), between 2 and 9 Gy (240 common genes) and between 0.5 and 9 Gy (48 common genes). As shown in Venn diagrams displayed in Fig. 2, some genes were deregulated in a unique way according to the specific dose delivered, suggesting a characteristic dose-dependent molecular response. A common gene list could represent the chance for cells to respond in a common way to irradiation, activating specific cell networks. Interestingly, 92 genes (Supplementary file 1) were deregulated after all the three doses used, and with the same expression trend. Thus, we decided to load this 92 gene signature into the Reactome tool in order to select the top five statistically relevant biological pathways. Results are provided in Table 7. In order to identify possible documented relationships between 92 microarray gene expression lists and some processes known to be involved in cell response to IR treatment, we used the PubMatrix V2.1 tool. In this way, lists of terms, such as protein names, can be assigned to a genetic, biological or clinical relevance in a flexible systematic fashion in order to confirm our assumptions. The common pathways activated in MCF10A, regardless of the used dose, involve networks related to inflammation, lipid metabolism and detoxification processes, which are not involved in death or cell fate balance, nor typically linked to the IR response.

**Table 4.** Top five molecular pathways of deregulated gene data sets of MCF10A, MCF7 and MDA-MB-231 cells treated with 0.5 Gy

		Pathway name	Genes found in GEP list	Entities (total)	*P*-value	FDR
**MCF10A**	1	Fatty acids	4	29	1.85E-2	7.21E-1
	2	Activation of the phototransduction cascade	3	19	2.84E-2	7.21E-1
	3	Relaxin receptors	2	8	3.14E-2	7.21E-1
	4	cGMP effects	3	20	3.24E-2	7.21E-1
	5	Glucagon-type ligand receptors	4	35	3.36E-2	7.21E-1
**MCF7**	1	Signal regulatory protein family interactions	3	16	2.01E-3	5.21E-1
	2	GABA synthesis	1	2	3.02E-2	5.96E-1
	3	ABC transporters in lipid homeostasis	2	18	3.16E-2	5.96E-1
	4	GABA synthesis, release, reuptake and degradation	2	19	3.48E-2	5.96E-1
	5	NR1D1 (REV-ERBA) represses gene expression	1	3	4.49E-2	5.96E-1
**MDA-MB-231**	1	ERBB2 activates PTK6 signaling	5	18	2.71E-3	8.39E-1
	2	ERBB2 regulates cell motility	5	19	3.41E-3	8.39E-1
	3	GRB2 events in ERBB2 signaling	5	20	4.23E-3	8.39E-1
	4	Hydroxycarboxylic acid-binding receptors	3	7	6.07E-3	8.39E-1
	5	Post-transcriptional silencing by small RNAs	3	7	6.07E-3	8.39E-1

**Table 5.** Top five molecular pathways of deregulated gene data sets of MCF10A, MCF7 and MDA-MB-231 cells treated with 2 Gy

		Pathway name	Genes found in GEP list	Entities (total)	*P*-value	FDR
**MCF10A**	1	Interleukin-4 and 13 signaling	23	212	1.01E-3	7.22E-1
	2	RUNX3 regulates immune response and cell migration	4	10	2.03E-3	7.22E-1
	3	RUNX1 regulates transcription of genes involved in differentiation of keratinocytes	4	11	2.85E-3	7.22E-1
	4	Transcriptional activation of p53-responsive genes	3	6	4.03E-3	7.22E-1
	5	Transcriptional activation of cell cycle inhibitor p21	3	6	4.03E-3	7.22E-1
**MCF7**	1	TP53 regulates transcription of death receptors and ligands	7	18	1.29E-6	8.42E-4
	2	TP53 regulates transcription of cell death genes	11	83	4.14E-5	1.35E-2
	3	Signal regulatory protein family interactions	5	18	2.08E-4	4.51E-2
	4	Interaction between L1 and ankyrins	6	33	4.66E-4	7.6E-2
	5	Transcriptional activation of cell cycle inhibitor p21	3	6	7.71E-4	8.33E-2
**MDA-MB-231**	1	Activation of kainate receptors upon glutamate binding	6	34	1.57E-2	8.76E-1
	2	RUNX1 regulates transcription of genes involved in differentiation of keratinocytes	3	11	2.72E-2	8.76E-1
	3	TNFs bind their physiological receptors	5	30	3.25E-2	8.76E-1
	4	MET activates STAT3	2	5	3.49E-2	8.76E-1
	5	Apoptosis-induced DNA fragmentation	3	13	4.13E-2	8.76E-1

**Table 6.** Top five molecular pathways of deregulated gene data sets of MCF10A, MCF7 and MDA-MB-231 cells treated with 9 Gy

		Pathway name	Genes found in GEP list	Entities (total)	*P*-value	FDR
**MCF10A**	1	Transcriptional activation of p53-responsive genes	4	6	4.02E-4	1.52E-1
	2	Transcriptional activation of cell cycle inhibitor p21	4	6	4.02E-4	1.52E-1
	3	RUNX1 regulates transcription of genes involved in differentiation of keratinocytes	5	11	4.37E-4	1.52E-1
	4	TP53 regulates transcription of genes involved in G1 cell cycle arrest	6	20	1.04E-3	2.7E-1
	5	RUNX3 regulates immune response and cell migration	4	10	2.6E-3	5.08E-1
**MCF7**	1	TP53 regulates transcription of death receptors and ligands	7	18	4.63E-6	3.67E-3
	2	TP53 regulates transcription of cell death genes	11	83	2.28E-4	8.99E-2
	3	Transcriptional activation of cell cycle inhibitor p21	3	6	1.35E-3	2.66E-1
	4	Transcriptional activation of p53-responsive genes	3	6	1.35E-3	2.66E-1
	5	Transcriptional regulation by TP53	29	486	4.89E-3	7.2E-1
**MDA-MB-231**	1	Interleukin-10 signaling	15	88	4.99E-3	8.85E-1
	2	Defective CHST6 causes MCDC1	4	9	5.94E-3	8.85E-1
	3	Interleukin-4 and -13 signaling	28	212	6.46E-3	8.85E-1
	4	ERBB2 regulates cell motility	5	19	1.82E-2	8.85E-1
	5	Apoptosis-induced DNA fragmentation	4	13	2.03E-2	8.85E-1

**Table 7.** Top five molecular pathways of selected gene signatures among MCF10A, MCF7 and MDA-MB-231 cells lines

	Pathway name	Genes found in GEP list	Entities (total)	*P*-value	FDR
**(A) Top five statistically relevant pathwaysof the MCF10A 92 gene signature**					
1	Eicosanoids	3	25	9.14E-4	1.9E-1
2	Fatty acids	3	29	1.4E-3	1.9E-1
3	Acrosome Reaction	1	1	7.42E-3	3.37E-1
4	Glutathione conjugation	3	67	1.41E-2	3.37E-1
5	HDL remodeling	2	24	1.41E-2	3.37E-1
**(B) Top five statistically relevant pathwaysof the MCF7 58 gene signature**					
1	Signal regulatory protein family interactions	3	16	1.03E-4	1.34E-2
2	GABA synthesis, release, reuptake and degradation	2	19	5.07E-3	3.3E-1
3	Synthesis of IP3 and IP4 in the cytosol	2	26	9.26E-3	3.52E-1
4	GABA synthesis	1	2	1.1E-2	3.52E-1
5	NR1D1 (REV-ERBA) represses gene expression	1	3	1.65E-2	3.97E-1
**(C) Top five statistically relevant pathways of the MDA-MB-231 265-gene signature**					
1	Binding of TCF/LEF:CTNNB1 to target gene promoters	2	10	1.89E-2	6.94E-1
2	Repression of WNT target genes	2	16	4.47E-2	6.94E-1
3	Glycogen metabolism	3	43	6.22E-2	6.94E-1
4	Gene and protein expression by JAK–STAT signaling after interleukin-12 stimulation	4	74	7.09E-2	6.94E-1
5	TFAP2 (AP-2) family regulates transcription of growth factors and their receptors	2	21	7.2E-2	6.94E-1

#### MCF7 breast cancer cell line

**Fig. 3.**
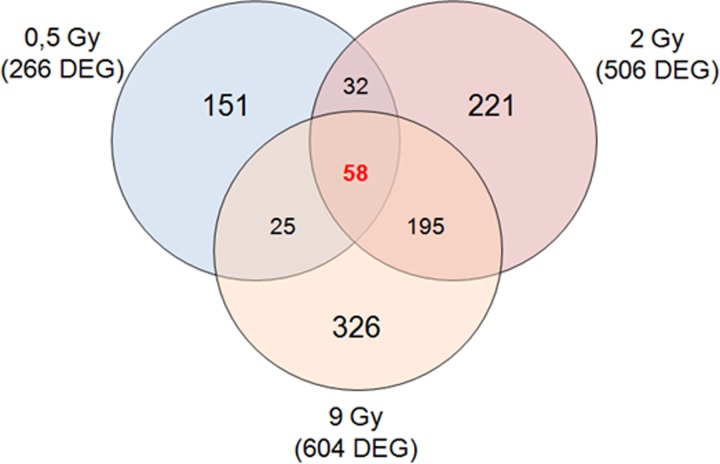
Venn diagram of microarray gene sets of MCF7 cells exposed to 0.5, 2 and 9 Gy of proton irradiation.

The MCF7 BC cell line was exposed to proton beam irradiation using 0.5, 2 and 9 Gy doses. As described above, the MCF7 cell line response to proton irradiation was analyzed 24 h post-irradiation using a two-color microarray-based gene expression approach. GEPs of the MCF7 cell line exposed to 0.5, 2 and 9 Gy revealed that multiple genes had significantly altered their expression levels, by ≥2-fold compared with the untreated MCF7 cells, used as reference group: MCF7 0.5 Gy, 266 DEGs (97 down-regulated and 169 up-regulated); MCF7 2 Gy, 506 DEGs (134 down-regulated and 291 up-regulated); and MCF7 9 Gy, 604 DEGs (158 down-regulated and 446 up-regulated) (Fig. 3).

Also in this case, up- and down-regulated transcripts were selected and grouped according to their involvement in specific biological pathways using integrated pathway enrichment analysis with Reactome, and the top significant pathways were analyzed for all the configurations assayed (Table 4, 5 and 6).

The result of this mapping revealed involvement of a set of factors controlling specific cellular processes, such as inflammation, response to drug, cell fate regulation and cellular trafficking in comparison with the reference sample. In addition, the GEP lists were also analyzed by Venn diagrams in order to identify overlapping deregulated genes among all the configuration assayed in this work, as shown in Fig. 3. In proton-treated MCF7 BC cells, some common genes were deregulated between 0.5 and 2 Gy (32 common genes), between 2 and 9 Gy (195 common genes) and between 0.5 and 9 Gy (25 common genes). Moreover, as displayed, 58 deregulated genes were common between all the three configurations analyzed (Supplementary file 1).

We speculated that this 58 gene signature could be responsible for the activation of intracellular mechanisms in response to radiation-induced stress. This molecular response appears not to be dependent on the specific dose delivered. Thus, we fed this gene signature into the Reactome tool, but no statistically relevant biological processes known to be related to cell response to radiation were selected (Table 7). These pathways involve GABA (γ-aminobutyric acid) metabolism, transduction signaling and gene expression regulation.

#### MDA-MB-231 metastatic breast cancer cell line

**Fig. 4.**
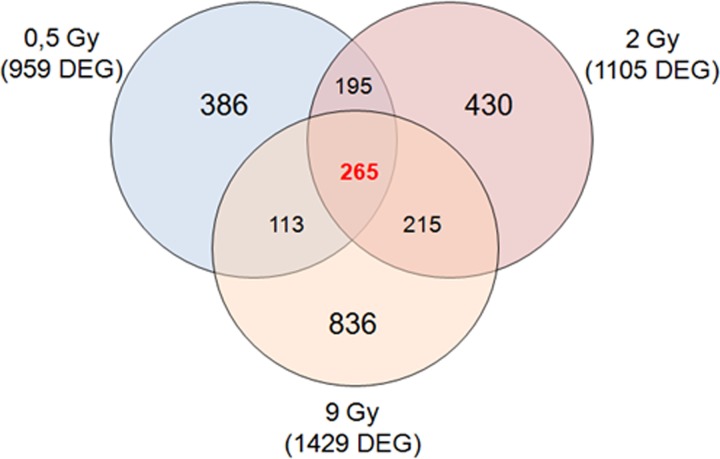
Venn diagram of microarray gene sets of MDA-MB-231 cells exposed to 0.5, 2 and 9 Gy of proton irradiation.

As described above for MCF10A and MCF7 cell lines, the same GEP approach with a two-color microarray-based gene expression analysis was performed for MDA-MB-231 cells 24 h post-irradiation. Comparative differential gene expression analysis revealed that multiple genes were significantly altered, by ≥2-fold, compared with untreated cells as follows: MDA-MB-231 0.5 Gy, 959 DEGs (266 down-regulated and 693 up-regulated); MDA-MB-231 2 Gy, 1105 DEGs (328 down-regulated and 777 up-regulated); and MDA-MB-231 9 Gy, 1429 DEGs (389 down-regulated and 1040 up-regulated) (Fig. 4).

Also in this case, an integrated pathway enrichment analysis was performed for all the configurations assayed. Summarized Reactome data regarding the top five pathways are displayed in Tables 4, 5 and 6. The result of this mapping revealed involvement of a set of factors controlling specific intracellular signaling belonging to ERBB2 pathway apoptosis, gene transcription, and inflammatory and anti-inflammatory pathways. In addition, the overlapping deregulated genes among all the configuration analyzed for MDA-MB-231 cells were highlighted by Venn diagrams, as shown in Fig. 4. In proton-treated MDA-MB-231 cells, some common genes were deregulated between 0.5 and 2 Gy (195 common genes); 2 and 9 Gy (215 common genes); and 0.5 and 9 Gy (113 common genes). As shown in Venn diagrams, 265 deregulated genes were common between all the three configurations assayed (Supplementary file 1), and we speculate that this gene signature could be responsible for the activation of intracellular mechanisms able overall to react to stress, such those induced by IR. Also in this case, we decided to load this gene signature into the Reactome tool which highlighted statistically relevant biological processes activated by proton exposure, known to be related to cell response to radiation, as displayed in Table 7. These are involved in gene expression regulation, WNT signalling, inflammation and glycogen metabolism.

### Dose-related gene signature of BC cell lines

**Fig. 5.**
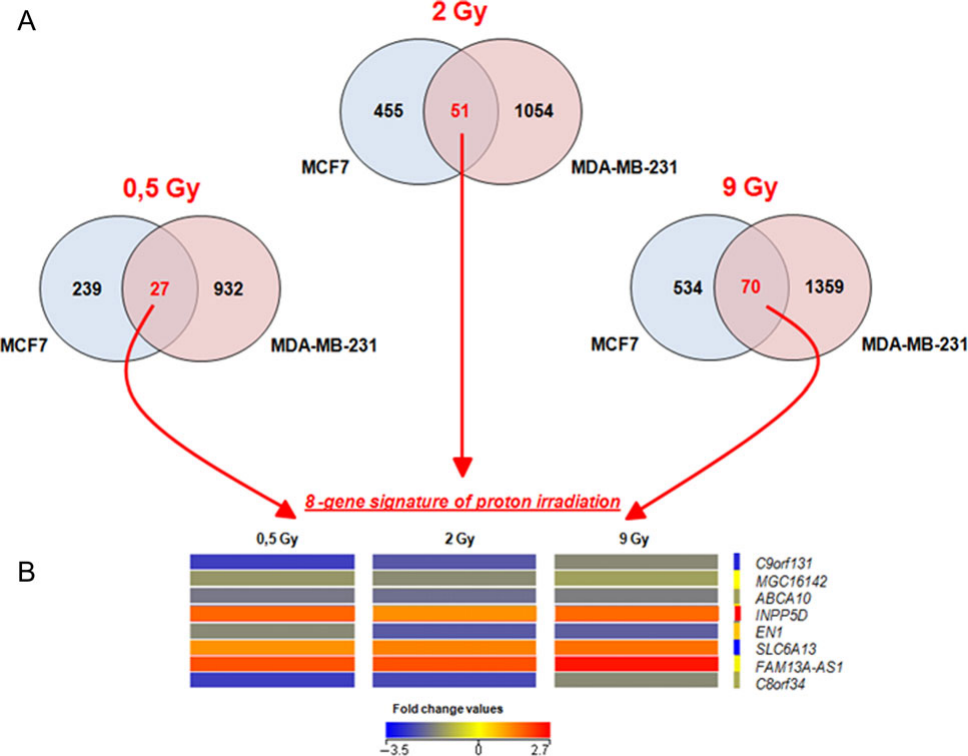
(**A**) 27–51 and 70 gene dose-related gene signature of BC cell lines exposed to 0.5, 2 and 9 Gy. (**B**) Heatmap of the 8 gene signature deregulated in BC cells after all three configuration doses. The individual values contained in a GEP were represented as colors according to their expression with respect to the control. Overall, down- and up-regulated genes after treatments were displayed using blue and red scales, as indicated in the key.

Finally, in order to evaluate the DEGs and cellular pathway deregulated in BC cells (MCF7 and MDA-MB-231), as a function of the delivered dose, we produced Venn diagrams as displayed in Fig. 5. Overall, common and unique genes were deregulated in BC cells after proton irradiation using the same dose. In particular, 27, 51 and 70 common genes were activated in BC cells after 0.5, 2 and 9 Gy of proton exposure, respectively. The eight genes shared between BC cells exposed to the three doses of proton irradiation used are as follows: chromosome 9 open reading frame 131 (C9orf131); uncharacterized protein MGC16142 (MGC16142); ATP-binding cassette, subfamily A (ABC1), member 10 (ABCA10); inositol polyphosphate-5-phosphatase (INPP5D); engrailed homeobox 1 (EN1); solute carrier family 6 (neurotransmitter transporter), member 13 (SLC6A13); AS1FAM13A antisense RNA 1 (FAM13A); and chromosome 8 open reading frame 34 (C8orf34).

Figure 4 displays the heatmaps of the hierarchical condition tree of the above-mentioned eight gene signatures related to proton irradiation cell response. As known, this is a graphical representation of data where the individual values contained in a GEP are represented as colors according to their expression with respect to the control. Overall, down- and up-regulated genes after treatment are displayed using blue and red scales, as indicated in the key.

Moreover, the 27, 51–70 and 8 gene signatures, were loaded into the Reactome tool in order to select the related pathways as shown in Table 8.

**Table 8.** Top five molecular pathways of BC selected gene signatures

	Pathway name	Genes found in GEP list	Entities (total)	*P*-value	FDR
**(A) Top five statistically relevant pathways of the 0.5 Gy 27 gene signature of BC cells**					
1	Reuptake of GABA	1	4	1.01E-2	2.71E-1
2	Digestion of dietary carbohydrate	1	10	2.51E-2	2.71E-1
3	PECAM1 interactions	1	12	0.03	2.71E-1
4	ABC transporters in lipid homeostasis	1	18	4.47E-2	2.71E-1
5	Ephrin signaling	1	19	4.71E-2	2.71E-1
**(B) Top five statistically relevant pathways of the 2 Gy 51 gene signature of BC cells**					
1	Interaction between L1 and ankyrins	3	33	3.09E-4	3.7E-2
2	Thyroxine biosynthesis	2	27	5.11E-3	3.07E-1
3	L1CAM interactions	3	129	1.42E-2	3.86E-1
4	Phase 0—rapid depolarisation	2	47	1.47E-2	3.86E-1
5	VEGF ligand–receptor interactions	1	8	3.08E-2	3.86E-1
**(C) Top- five statistically relevant pathways of the 9 Gy 70 gene signature of BC cells**					
1	Interleukin-35 signaling	2	16	3.65E-3	4.3E-1
2	Extracellular matrix organization	6	329	9.89E-3	4.3E-1
3	Thyroxine biosynthesis	2	27	0.01	4.3E-1
4	Integrin cell surface interactions	3	86	1.23E-2	4.3E-1
5	Amine-derived hormones	2	56	3.88E-2	4.42E-1

Finally, we decided to confirm and validate the involvement of genes belonging to the above-mentioned signatures, using an *in silico* approach by PubMatrix, as described in the literature and also by our group [31]. In this way, lists of terms, such as gene names, were assigned to genetic, biological or clinical relevance in a flexible systematic fashion in order to confirm our assumptions. Bibliographic relationships between proteins and selected queries such as IR, radiation, cancer, BC, electron, proton and inflammation were analyzed in order to understand the data and to draw useful conclusions reported below (Supplementary files 3, 4, 5 and 6).

## DISCUSSION

There is a substantial lack of data regarding cancer cell and molecular responses induced by proton irradiation, which prompted this work. Moreover, the possibility that proton therapy may be used to treat BC led us to study this topic in breast cell lines. The identification of gene signatures linked to specific radiation regimens, as reported in this work, could be helpful for the understanding of the molecular mechanisms linked with the treatment efficacy, allowing the introduction of biological features in RT planning. In this sense, for the first time to our knowledge, we have performed molecular analyses of BC cells exposed to different doses of proton irradiation, highlighting gene expression signatures related to specific cell lines and proton irradiation configurations. Data collected here confirm that radiation effects on cells are heterogeneous and appear to act in a cell line-dependent manner. This may in turn lead to an individualized form of treatment with a significant predictive value.

Dose–response effects were analyzed on the non-tumorigenic (MCF10A) and tumorigenic BC (MCF7 and MDA-MB-231) cell lines with different aggressive phenotypes. The SFs obtained showed different cellular radiosensitivity; in particular, the MCF7 cells revealed greater sensitivity to proton irradiation than MDA-MB-231 and MCF10A cells, which were more radioresistant (Fig. 1).

We performed Luminex and cDNA microarray gene expression analyses to study molecules and biological processes activated by exposure to charged particle beams (using 0.5, 2 and 9 Gy of protons) on the three breast cell lines. Considering that IR induces significant effects on immune system modulation able to drive the survival/cell death balance and senescence process, we first studied the cytokine, chemokine and growth factor profiles induced in MCF10A, MCF7 and MDA-MB-231 cell lines by proton irradiation (Tables 1–3; Supplementary file 2). These molecules are secreted by treated normal epithelial and residual BC cells in the tumor microenvironment, driving the tissue IR response by autocrine and paracrine mechanisms.

Overall, we investigated the time and dose dependence of cytokine signatures for the three cell lines tested, highlighting some differences that we think may also be justified by the different aggressive phenotypes of the three BC cell lines. Indeed, while MCF10A cells are strongly affected by the dose effect, since strong quantitative differences are observable between low (0.5 and 2 Gy) and high (9 Gy) doses for most of the molecules assayed (see Supplementary file 2), the MDA-MB-231 cells suffer much less from the dose effect, since no significant quantitative differences are observable between the low doses and the 9 Gy dose. In contrast, for MCF7 cells, an irregular pattern is observed for six out of eight tested molecules, as only IL-6 and IL-8 appear to be produced in a time- and dose-dependent manner. In particular, MCF10A cells subjected to 9 Gy show a polynomial-type increasing trend of inflammatory molecules, TH1-type, TH2-type, chemokines and growth factors, although the two chemokines IL-8 and MCP-1 have been identified as early irradiation markers as their relative increase is observable as early as 48 h post-treatment, regardless of the dose delivered within the same time. These two molecules (IL-8 and MCP-1) and IL-6 are produced in a time- and dose-dependent manner in MCF7 cells, with peaks observable at 72 h post-irradiation.

The immune response profile of MDA-MB-231 cells to irradiation was characterized by a stronger activation, in a time-dependent manner and regardless of dose delivered, of 11 out 17 of the immunological factors analyzed, compared with to MCF10A and MCF7 cells. Summarizing, MDA-MB-231 cells showed the strongest potentially pro-inflammatory profile compared with the other cell lines analyzed. The inflammatory network seems to be unbalanced as IL-10, the main anti-inflammatory cytokine, is not expressed and the IL-4/IL-13 up-regulation is rather limited. Moreover, the observed strong IFN-γ up-regulation at low and high doses denotes a TH1-type response activation.

Considering the three immunological profiles, IL-6, IL-8 and MCP-1 could be considered candidate biomarkers of proton irradiation of mammary epithelial cells.

Interestingly, these results are comparable with those obtained by our group for the same cell lines following electron beams [17]. However, the immunological profiles of the three different cell lines do not seem to exert a direct role on cell death increasing during the time window of this *in vitro* experiment. On the other hand, it could be speculated that the release of these molecules can play a different and specific paracrine role in each patient’s tumor microenvironment, generating individual long-term effects on tumor progression and post-treatment sequelae, e.g. local or systemic inflammation, fibrosis or cell senescence.

Moreover, as graphically displayed, some genes were specifically deregulated according to the dose delivered (dose-dependent gene lists), whereas other genes (cell-dependent gene list) were commonly deregulated after all the three doses delivered and were probably linked to BC-specific features (Figs 2, 3 and 4). All the gene signatures selected in this work are available in Supplementary file 1, whereas Tables 4, 5 and 6 displays the top five statistically significant pathways activated in the three cell lines studied in response to 0.5, 2 and 9 Gy, respectively.

In MCF10A cells, a 92 common gene list (Fig. 2) has been identified to sustain the molecular response to irradiation regardless of the used dose, which participate in the activation of pathways related to inflammation, lipid metabolism and detoxification processes, listed in Table 7A.

Regarding MCF7 BC cells, the TP53 pathway appears to drive the signaling controlling the response to radiation. This finding is in line with our previous investigations on the MCF7 molecular response to radiation using electron beams [22]. In these cells, a 58 gene signature is activated regardless of the dose used, and the genes sustain general pathways, GABA metabolism, transduction signaling and gene expression regulation, which appear not to be directly related to such a stress response.

Moreover, for the MDA-MB-231 BC cell line, we also selected unique and common gene signatures linked to the dose delivered (Tables 4, 5 and 6). Interestingly, IR is able to activate ERBB2 signaling in the MDA-MB-231 cells; nonetheless, they are HER2– and classified as triple-negative cells. Furthermore, the activation of inflammatory-related pathways justifies the great capacity of MDA-MB-231 cells to release inflammatory molecules in the tumor microenvironment, as highlighted in this work. Moreover, this behaviour has been previously noted by our group even in response to irradiation using electron beams, a sign that activation of inflammation is typical of the IR response for these cells [12]. Through inflammation, these cells control cell fate balance, senescence mechanisms, angiogenesis and cell migration, explaining their aggressive phenotype and the resistance to the treatments, driving the activation of intracellular mechanisms able to react to stress and known to be related to the cell response to radiation, as displayed in Table 7C. These are involved in regulation of gene expression, WNT signaling, inflammation and glycogen metabolism.

We also decided to analyze, for the first time to our knowledge, proton dose-related gene signatures, specific for breast cancer cells (i.e. MCF7 and MDA-MB-231). Interestingly, we selected 27, 51 and 70 genes that were activated in BC cells after 0.5, 2 and 9 Gy, respectively (Table 8). Moreover, in order to select specific biomarkers of proton cell response, we selected eight common deregulated genes (8 gene signature) shared among the 27, 51 and 70 gene signatures selected and mentioned above (Fig. 4).

All genes belonging to these signatures were also analyzed by the PubMatrix tool, in order to search for biological interpretation through NCBI literature of data obtained from microarray analysis.

In particular, we speculate that these genes could represent specific biomarkers of proton breast cell response. Among these genes, no information about the involvement of C9orf131, MGC16142, FAM13A and C8orf3 genes in the radiation cell response (proton or other types) is now available in the literature, which requires further investigation

On the other hand, limited but promising data were collected for the other genes of the 8 gene signature. In particular, the ABCA10 gene codes for a member of the superfamily of ATP-binding cassette (ABC) transporters, often overexpressed in several tumors and responsible for the traffic of a wide variety of xenobiotics, lipids and metabolic products across the cell membranes. These proteins were also described as implicated in multidrug resistance, but no information is available regarding their hypothetical role in radioresistance processes [32, 33].

The INPP5D gene is a member of the inositol polyphosphate-5-phosphatase (INPP5) family regulated by AKT signaling and recently described as involved in the apoptotic process, also during hypoxia conditions independently of p53 activation [34]. As has been well described, AKT is able to regulate the survival/death balance, in response to radiation [35]. In addition, AKT activation in hypoxic regions of breast tumors has been linked with poor patient prognosis, whereas AKT inhibition may improve radiotherapy response in p53-deficient tumors, and for this purpose further studies are needed to evaluate the role of INPP5D in the radioresistance process.

The EN1 gene is involved in the development of the central nervous system, and recently was described as a prosurvival transcription factor in the basal-like BC type. It is also described as exclusively overexpressed in some extremely aggressive cancers, also associated with hypoxia, inflammation and high leukocyte infiltration [36]. However, no information regarding its role and radiation exposure is available.

The SLC6A13 gene encodes a neurotransmitter transporter involved in the uptake of δ-aminolevulinic acid (ALA). ALA-induced protoporphyrin accumulation is a strategy widely used in cancer treatment. Its expression was found in some cancer cell lines and can play a role in enhancing the accumulation of ALA-induced protoporphyrin [37]. Additional studies are needed in order to identify its role in the radiation cell response.

Overall, data from immunological signatures and the top five lists of GEP profiling seem to intersect at some points; nonetheless, transcriptional and translational levels are not always coincident and the time windows analyzed are different. In particular, immunological pathways which varied following radiation among the top five lists analyzed are: (i) Interleukin 4 and 13 signaling with 23 genes varying in MCF10 exposed to 2 Gy; and (ii) the two anti-inflammatory pathways: Interleukin 10 and Interleukin 4 and 13 signaling, with 15 and 28 genes varying in MDA-MB-231 exposed to 9 Gy. In addition, in MDA-MB-231, the ‘Gene and protein expression by Jak–STAT signalling after IL-12 stimulation’ is commonly different among all the dose configurations analyzed.

In MCF7 cells, the GEP profiling does not involve immunological pathways, justifying the poor secretion levels of immunological proteins, whereas in MCF10 and MDA-MB-231 cells the transcriptional activation of an anti-inflammatory response sustained by the IL-4/IL-13 loop is still observable 24 h post-treatment, to balance inflammation generated by IR. Furthermore, the conspicuous IFN-γ release 48–72 h post-treatment by MDA-MB-231 cells exposed to all the doses is fully explained by the activation of ‘Jak–STAT signalling after IL-12 stimulation’ 24 h post-irradiation.

In conclusion, as previously described by several authors and also by our group [12, 13, 38], radiation effects on cells are heterogeneous and appear to act in a cell line-dependent manner, and this behavior is also confirmed in response to proton beam irradiation.

## CONCLUSIONS

This work highlighted the molecular response to proton irradiation of three breast cell lines (the tumorigenic MCF7 and MDA-MB-231 and the non tumorigenic MCF10A), in terms of GEP and secretion of immunological molecules. The three cell lines studied demonstrated differential GEP/cytokine profiles after proton irradiation, showing a cell line- and dose-dependent type of response to radiation. The immunological signatures are dependent on the dose delivered (low doses of 0.5 and 2 Gy vs a high dose of 9 Gy) in MCF10A and MCF7 cell lines, whereas MDA-MB-231 cells show a strong pro-inflammatory expression profile regardless of the dose. We selected a specific 8 gene signature which represents a list of common genes deregulated in response to the doses used and in all the cell lines analyzed in this work that in our opinion need more investigation. It can be envisaged that our data could be useful in the definition of personalized proton therapy protocols in combination with targeted therapies dedicated to breast cancer.

## Supplementary Material

Supplementary Data

Supplementary Data

Supplementary Data

Supplementary Data

Supplementary Data

Supplementary Data
