# Supplementary material for: Proton-irradiated breast cells: molecular points of view
Source: J Radiat Res. 2019 May 28;60(4):451–65. doi: 10.1093/jrr/rrz032 (PMC6640903; doi:10.1093/jrr/rrz032)
Supplement: Supplementary Data [file rrz032_additional_file_1.pdf]

	92-gene signature of MCF10A cells proton treated	
	**GeneSymbol**	**Description**
**1**	ACR	Acrosin
**2**	ADH1B	Alcohol dehydrogenase 1B (class I), beta polypeptide
**3**	C1orf189	Chromosome 1 open reading frame 189
**4**	C6orf10	Chromosome 6 open reading frame 10
**5**	C8orf34	Chromosome 8 open reading frame 34
**6**	C9orf131	Chromosome 9 open reading frame 131
**7**	CENPL	Centromere protein L
**8**	CNGA1	Cyclic nucleotide gated channel alpha 1
**9**	CXCL1	Chemokine (C-X-C motif) ligand 1
**10**	DPY19L2	Dpy-19-like 2
**11**	EDNRB	Endothelin receptor type B
**12**	ERVW-1	Endogenous retrovirus group W, member 1
**13**	FAM154A	Family with sequence similarity 154, member A
**14**	GPR37L1	G protein-coupled receptor 37 like 1
**15**	HPGD	Hydroxyprostaglandin dehydrogenase 15-
**16**	IL26	Interleukin 26
**17**	INIP	Chromosome 9 open reading frame 80
**18**	KCNAB3	Potassium channel, voltage gated subfamily A regulatory beta subunit 3
**19**	L1CAM	L1 cell adhesion molecule
**20**	LINC01117	Long intergenic non-protein coding RNA 1117
**21**	MIR7515HG	MIR7515 host gene
**22**	NREP	Neuronal regeneration related protein
**23**	ODAM	Odontogenic, ameloblast asssociated
**24**	OLFM4	Olfactomedin 4
**25**	PAX6	Paired box 6 (PAX6), transcript variant 1
**26**	RFX4	Regulatory factor X, 4 (influences HLA class II expression)
**27**	SDHC	Succinate dehydrogenase complex, subunit C, integral membrane protein, 15kDa
**28**	SHC3	SHC (Src homology 2 domain containing) transforming protein 3 (SHC3)
**29**	SLC23A3	Solute carrier family 23, member 3
**30**	SLC26A7	Solute carrier family 26 (anion exchanger), member 7
**31**	SLC5A10	Solute carrier family 5 (sodium/sugar cotransporter), member 10
**32**	TCEAL7	Transcription elongation factor A (SII)-like 7
**33**	TMEM132B	Transmembrane protein 132B
**34**	AGAP2	ArfGAP with GTPase domain, ankyrin repeat and PH domain 2
**35**	ASPA	Aspartoacylase
**36**	ATOH7	Atonal homolog 7
**37**	CD274	CD274 molecule
**38**	CEP44	Centrosomal protein 44kDa
**39**	CYP4F2	Cytochrome P450, family 4, subfamily F, polypeptide 2
**40**	DQX1	DEAQ box RNA-dependent ATPase 1
**41**	EPHA10	EPH receptor A10
**42**	ERICH6-AS1	ERICH6 antisense RNA 1
**43**	FAM13A-AS1	FAM13A antisense RNA 1
**44**	FAM175A	Family with sequence similarity 175, member A
**45**	FBLL1	Fibrillarin-like 1
**46**	FCRLA	Fc receptor-like A
**47**	FGF1	Fibroblast growth factor 1 (acidic)
**48**	FOXA2	Forkhead box A2
**49**	GJC3	Gap junction protein, gamma 3, 30,2kDa
**50**	GPR1	G protein-coupled receptor 1
**51**	GSTA2	Glutathione S-transferase alpha 2
**52**	HERC6	HECT and RLD domain containing E3 ubiquitin protein ligase family member 6
**53**	HLA-G	cDNA FLJ36159 fis, clone TESTI2025791, highly similar to HLA CLASS I HISTOCOMPATIBILITY ANTIGEN

**54**	HMMR-AS1	HMMR antisense RNA 1
**55**	HOXC11	Homeobox C11
**56**	KCNMA1	Potassium channel, calcium activated large conductance subfamily M alpha, member 1
**57**	KDM4D	Lysine (K)-specific demethylase 4D
**58**	KIAA1549L	KIAA1549-like
**59**	KITLG	KIT ligand (KITLG), transcript variant b
**60**	LAMA1	Laminin, alpha 1
**61**	LINC00515	Long intergenic non-protein coding RNA 515
**62**	LIPH	lipase, member H
**63**	lnc-PBX1-2	LNCipedia lincRNA (lnc-PBX1-2), lincRNA
**64**	lnc-SLC30A7-2	cDNA FLJ11489 fis
**65**	lnc-XRCC6-1	LNCipedia lincRNA
**66**	LOC100507140	Uncharacterized
**67**	MAGEL2	MAGE-like 2
**68**	MARCH3	Membrane-associated ring finger (C3HC4) 3, E3 ubiquitin protein ligase
**69**	MUC16	Mucin 16, cell surface associated
**70**	NLRP3	NLR family, pyrin domain containing 3
**71**	NPY6R	Neuropeptide Y receptor Y6 (pseudogene)
**72**	OR2W3	Olfactory receptor, family 2, subfamily W, member 3
**73**	OXR1	Oxidation resistance 1
**74**	PKD1L2	Polycystic kidney disease 1-like 2 (gene/pseudogene)
**75**	PLEKHG1	Pleckstrin homology domain containing, family G
**76**	PLTP	Phospholipid transfer protein
**77**	PRSS23	Protease, serine, 23
**78**	PRSS23	Protease, serine, 23
**79**	RRAD	Ras-related associated with diabetes
**80**	SCEL	Sciellin
**81**	SIAE	Sialic acid acetylesterase
**82**	SLC6A6	Solute carrier family 6 (neurotransmitter transporter), member 6
**83**	SLC8A3	Solute carrier family 8 (sodium/calcium exchanger), member 3
**84**	SNX29	Sorting nexin 29
**85**	SP5	Sp5 transcription factor
**86**	SPRR2G	Small proline-rich protein 2G
**87**	SRCIN1	SRC kinase signaling inhibitor 1
**88**	TGM2	Transglutaminase 2
**89**	TRIML2	Tripartite motif family-like 2
**90**	TTLL7	Tubulin tyrosine ligase-like family member 7
**91**	UBR4	Ubiquitin protein ligase E3 component n-recognin 4
**92**	YEATS2	cDNA FLJ33401 fis

	58-gene signature of MCF10A cells proton treated	
	**GeneSymbol**	**Description**
**1**	ABCA10	ATP-binding cassette, sub-family A (ABC1), member 10
**2**	ARMC4	Armadillo repeat containing 4
**3**	BRINP3	Bone morphogenetic protein/retinoic acid inducible neural-specific 3
**4**	C8orf34	Chromosome 8 open reading frame 34
**5**	C9orf131	Chromosome 9 open reading frame 131
**6**	CALD1	Caldesmon 1
**7**	CCDC113	Coiled-coil domain containing 113
**8**	COLQ	Collagen-like tail subunit (single strand of homotrimer) of asymmetric acetylcholinesterase
**9**	COX18	COX18 cytochrome c oxidase assembly factor
**10**	CTAGE5	CTAGE family
**11**	DNAH2	Dynein, axonemal, heavy chain 2
**12**	DNAJC21	DnaJ (Hsp40) homolog, subfamily C, member 21
**13**	EDNRB	Endothelin receptor type B
**14**	EN1	Engrailed homeobox 1
**15**	EXT1	Exostosin glycosyltransferase 1
**16**	FAM13A-AS1	FAM13A antisense RNA 1
**17**	FAM223A	Family with sequence similarity 223, member A
**18**	GAD1	Glutamate decarboxylase 1
**19**	GPR37L1	G protein-coupled receptor 37 like 1
**20**	INPP5D	Inositol polyphosphate-5-phosphatase
**21**	ITPKB	Inositol-trisphosphate 3-kinase B
**22**	JAZF1	JAZF zinc finger 1
**23**	LAPTM5	Lysosomal protein transmembrane 5
**24**	LINC00319	long intergenic non-protein coding RNA 319
**25**	lnc-PPIAL4G-6	LNCipedia lincRNA
**26**	lnc-SUSD1-1	cDNA FLJ31796 fis
**27**	LOC100507140	Uncharacterized LOC100507140
**28**	LOC284561	PREDICTED: uncharacterized LOC284561
**29**	LOC728175	Uncharacterized LOC728175
**30**	MGC16142	Uncharacterized protein MGC16142
**31**	MPL	MPL proto-oncogene, thrombopoietin receptor
**32**	MYO16	Myosin XVI
**33**	NAT8	N-acetyltransferase 8 (GCN5-related, putative)
**34**	NCOR1	Nuclear receptor corepressor 1
**35**	NREP	Neuronal regeneration related protein
**36**	OR2W3	Olfactory receptor, family 2, subfamily W, member 3
**37**	POU5F2	POU domain class 5, transcription factor 2
**38**	RIBC1	RIB43A domain with coiled-coils 1
**39**	RNF183	Ring finger protein 183
**40**	RNF19B	Ring finger protein 19B
**41**	SCGN	Secretagogin, EF-hand calcium binding protein
**42**	SEC62	SEC62 homolog
**43**	SERPINC1	Serpin peptidase inhibitor, clade C (antithrombin), member 1
**44**	SIRPA	Signal-regulatory protein alpha
**45**	SLC5A12	Solute carrier family 5 (sodium/monocarboxylate cotransporter), member 12
**46**	SLC6A13	Solute carrier family 6 (neurotransmitter transporter), member 13
**47**	SMOC1	SPARC related modular calcium binding 1
**48**	SNED1	Sushi, nidogen and EGF-like domains 1
**49**	SP5	Sp5 transcription factor
**50**	SPATA6	Spermatogenesis associated 6
**51**	TGM4	Transglutaminase 4
**52**	TMEM232	Transmembrane protein 232
**53**	TRIM22	Tripartite motif containing 22

**54**	TRIM49	Tripartite motif containing 49
**55**	TRIM63	Tripartite motif containing 63, E3 ubiquitin protein ligase
**56**	TTC30A	Tetratricopeptide repeat domain 30A
**57**	TTYH2	Tweety family member 2
**58**	XG	Xg blood group

	265-gene signature of MCF10A cells proton treated	
	**GeneSymbol**	**Description**
**1**	ABCA10	ATP-binding cassette, sub-family A (ABC1), member 10
**2**	ACAN	Aggrecan (ACAN), transcript variant X1
**3**	ACER3	Alkaline ceramidase
**4**	ACR	Acrosin
**5**	ACSBG2	Acyl-CoA synthetase bubblegum family member 2
**6**	ACTA1	Actin, alpha 1, skeletal muscle
**7**	ADAM21	ADAM metallopeptidase domain 21
**8**	ADAMTS19	ADAM metallopeptidase with thrombospondin type 1 motif, 19
**9**	ADAMTSL3	ADAMTS-like 3
**10**	AGBL2	ATP/GTP binding protein-like 2
**11**	ANKDD1A	Ankyrin repeat and death domain containing 1A
**12**	ANKRD20A5P	Ankyrin repeat domain 20 family, member A5, pseudogene
**13**	ANKRD26	Ankyrin repeat domain 26
**14**	ARHGAP8	Rho GTPase activating protein 8
**15**	ARHGEF7	Rho guanine nucleotide exchange factor (GEF) 7
**16**	ATF7IP2	Activating transcription factor 7 interacting protein 2
**17**	ATP2A1	ATPase, Ca++ transporting, cardiac muscle, fast twitch 1
**18**	ATPAF1	ATP synthase mitochondrial F1 complex assembly factor 1
**19**	AXIN2	Conductin
**20**	BAI3	Brain-specific angiogenesis inhibitor 3
**21**	BAIAP2L2	BAI1-associated protein 2-like 2
**22**	BMPR1B	bone morphogenetic protein receptor, type IB
**23**	C10orf82	Chromosome 10 open reading frame 82
**24**	C11orf52	Chromosome 11 open reading frame 52
**25**	C15orf27	Chromosome 15 open reading frame 27
**26**	C15orf59-AS1	Chromosome 15 open reading frame 59
**27**	C1orf189	Chromosome 1 open reading frame 189
**28**	C1S	Complement component 1, s subcomponent
**29**	C6orf10	Chromosome 6 open reading frame 10
**30**	C8orf34	Chromosome 8 open reading frame 34
**31**	C9orf131	Chromosome 9 open reading frame 131
**32**	CALD1	Caldesmon 1
**33**	CAMK1G	Calcium/calmodulin-dependent protein kinase IG
**34**	CCDC108	Coiled-coil domain containing 108
**35**	CCDC126	Coiled-coil domain containing 126
**36**	CCDC36	Coiled-coil domain containing 36
**37**	CCT8	Chaperonin containing TCP1, subunit 8 (theta)
**38**	CD226	CD226 molecule
**39**	CDH13	Cadherin 13
**40**	CENPL	Centromere protein L
**41**	CLEC2D	C-type lectin domain family 2, member D
**42**	CLMN	Calmin (calponin-like, transmembrane)
**43**	COL20A1	Collagen, type XX, alpha 1
**44**	CPLX2	Complexin 2
**45**	CRTAM	Cytotoxic and regulatory T cell molecule
**46**	CRYM	Crystallin, mu
**47**	CTSO	Cathepsin O
**48**	CX3CL1	Chemokine (C-X3-C motif) ligand 1
**49**	CYBB	Cytochrome b-245, beta polypeptide
**50**	CYP2U1	Cytochrome P450, family 2, subfamily U, polypeptide 1
**51**	CYP4X1	Cytochrome P450, family 4, subfamily X, polypeptide 1
**52**	DEFB123	Defensin, beta 123
**53**	DIP2B	DIP2 disco-interacting protein 2 homolog B

**54**	DNER	Delta/notch-like EGF repeat containing
**55**	DOC2B	Double C2-like domains, beta
**56**	DOPEY1	Dopey family member 1
**57**	DUOX2	Dual oxidase 2
**58**	ECSCR	Endothelial cell surface expressed chemotaxis and apoptosis regulator
**59**	EIF4E	Eukaryotic translation initiation factor 4E
**60**	ELOVL7	ELOVL fatty acid elongase 7
**61**	EME2	Essential meiotic structure-specific endonuclease subunit 2
**62**	EN1	Engrailed homeobox 1
**63**	EPB41L5	Erythrocyte membrane protein band 4,1 like 5]
**64**	EPM2A	Epilepsy, progressive myoclonus type 2A, Lafora disease (laforin)
**65**	EPPK1	EPPK1 mRNA for epiplakin 1, complete cds,
**66**	ERBB2	Erb-b2 receptor tyrosine kinase 2
**67**	ERMAP	Erythroblast membrane-associated protein
**68**	F13B	Coagulation factor XIII, B polypeptide
**69**	FAM13A-AS1	FAM13A antisense RNA 1
**70**	FAM175A	Family with sequence similarity 175, member A
**71**	FAM198A	Family with sequence similarity 198, member A
**72**	FAM19A2	Family with sequence similarity 19 (chemokine (C-C motif)-like), member A2
**73**	FAM65C	mRNA for FLJ00360 protein
**74**	FAM71D	Family with sequence similarity 71, member D
**75**	FAM86B3P	Family with sequence similarity 86, member B3
**76**	FAM90A7P	Family with sequence similarity 90, member A7
**77**	FBLL1	Fibrillarin-like 1
**78**	FFAR4	Free fatty acid receptor 4
**79**	FIGN	Fidgetin
**80**	FLJ36777	Uncharacterized LOC730971
**81**	FMNL3	Formin-like 3
**82**	FOSB	FBJ murine osteosarcoma viral oncogene homolog B
**83**	FOXA1	Forkhead box A1
**84**	FRMD8P1	FERM domain containing 8 pseudogene 1
**85**	FUBP1	Far upstream element (FUSE) binding protein 1
**86**	GAREML	GRB2 associated, regulator of MAPK1-like
**87**	GARNL3	GTPase activating Rap/RanGAP domain-like 3
**88**	GJC3	Gap junction protein, gamma 3
**89**	GPR52	G protein-coupled receptor 52
**90**	GPR64	G protein-coupled receptor 64
**91**	GRIK4	Glutamate receptor, ionotropic, kainate 4
**92**	HAND1	Heart and neural crest derivatives expressed 1
**93**	HHIP	Hedgehog interacting protein
**94**	HIPK4	Homeodomain interacting protein kinase 4
**95**	HIST1H1D	Histone cluster 1, H1d
**96**	HLA-DOA	Major histocompatibility complex, class II, DO alpha
**97**	HLA-DOA	Major histocompatibility complex, class II, DO alpha
**98**	HOXD9	Homeobox D9
**99**	HSH2D	Hematopoietic SH2 domain containing
**100**	HTR3E	5-hydroxytryptamine (serotonin) receptor 3E, ionotropic
**101**	ID2	Inhibitor of DNA binding 2, dominant negative helix-loop-helix protein
**102**	IGFN1	Immunoglobulin-like and fibronectin type III domain containing 1
**103**	IL17A	Interleukin 17A
**104**	INPP5D	Inositol polyphosphate-5-phosphatase, 145kDa
**105**	IRF4	Interferon regulatory factor 4
**106**	IVL	Involucrin
**107**	KCNAB3	Potassium channel, voltage gated subfamily A regulatory beta subunit 3
**108**	KIAA1549L	KIAA1549-like
**109**	KNG1	Kininogen 1

**110**	KRTAP13-4	Keratin associated protein 13-4
**111**	KRTAP19-1	Keratin associated protein 19-1
**112**	LDLRAD3	Low density lipoprotein receptor class A domain containing 3
**113**	LGALS9	Lectin, galactoside-binding, soluble, 9
**114**	LINC00174	Long intergenic non-protein coding RNA 174
**115**	LINC00421	Long intergenic non-protein coding RNA 421
**116**	LINC00442	Long intergenic non-protein coding RNA 442
**117**	LINC00896	Long intergenic non-protein coding RNA 896
**118**	LIPG	Lipase, endothelial
**119**	lnc-AC092031,1-1	LNCipedia lincRNA
**120**	lnc-ADA-1	601109106F1 NIH_MGC_16
**121**	lnc-CDKN2C-1	LNCipedia lincRNA
**122**	lnc-CELA3A-2	full length insert cDNA clone ZD58F06
**123**	lnc-GGCT-1	ALU4_HUMAN (P39191) Alu subfamily SB2 sequence contamination warning entry, partial (40%)
**124**	lnc-RNF219-3	cDNA FLJ11514 fis, clone HEMBA1002229
**125**	lnc-RNF39-4	non-protein coding RNA 171, mRNA (cDNA clone IMAGE:5167697)
**126**	lnc-RP11-298P3,4,1-1	Novel human gene mapping to chomosome 13
**127**	lnc-SYT16-1	cDNA FLJ31085 fis, clone IMR321000037
**128**	lnc-TMC5-1	Aj09f01,s1 Soares_parathyroid_tumor_NbHPA cDNA clone 1389817 3'
**129**	LOC100128644	Clone DNA147258 LMNE6487
**130**	LOC100129781	Uncharacterized LOC100129781
**131**	LOC100130920	cDNA FLJ36320 fis, clone THYMU2005480
**132**	LOC100133616	cDNA FLJ35883 fis, clone TESTI2008929
**133**	LOC100505824	cDNA clone IMAGE:5297975
**134**	LOC100506472	Uncharacterized LOC100506472
**135**	LOC100506538	Uncharacterized LOC100506538
**136**	LOC102725134	Uncharacterized LOC102725134
**137**	LOC200726	hCG1657980
**138**	LOC344887	NmrA-like family domain containing 1 pseudogene
**139**	LOC344887	NmrA-like family domain containing 1 pseudogene
**140**	LRG1	leucine-rich alpha-2-glycoprotein 1
**141**	LYZ	Lysozyme
**142**	MAGEL2	MAGE-like 2
**143**	MBP	Myelin basic protein
**144**	MED12L	Mediator complex subunit 12-like
**145**	MEGF10	Multiple EGF-like-domains 10
**146**	MGA	mRNA; cDNA DKFZp667M0810
**147**	MGC16142	Uncharacterized protein MGC16142
**148**	MIAT	Myocardial infarction associated transcript
**149**	MIR7515HG	MIR7515 host gene
**150**	MMRN2	Multimerin 2
**151**	MSN	Moesin
**152**	MYH6	Myosin, heavy chain 6, cardiac muscle, alpha
**153**	MYO5A	Myosin VA (heavy chain 12, myoxin)
**154**	NECAB1	N-terminal EF-hand calcium binding protein 1
**155**	NOL4L	Nucleolar protein 4-like
**156**	NPR3	Natriuretic peptide receptor 3
**157**	NRCAM	Neuronal cell adhesion molecule
**158**	NRN1	Neuritin 1
**159**	NTSR1	Neurotensin receptor 1
**160**	OR10C1	Olfactory receptor, family 10, subfamily C, member 1
**161**	OR10H1	Olfactory receptor, family 10, subfamily H, member 1
**162**	ORM2	Orosomucoid 2
**163**	OSR1	Odd-skipped related transciption factor 1
**164**	OVOL3	Ovo-like zinc finger 3
**165**	OXR1	Oxidation resistance 1

**166**	PACSIN1	Potein kinase C and casein kinase substrate in neurons 1
**167**	PALMD	Palmdelphin
**168**	PARP14	Poly (ADP-ribose) polymerase family, member 14
**169**	PAX6	Paired box 6
**170**	PCDH1	Protocadherin 1
**171**	PCDH15	Protocadherin-related 15
**172**	PCDH18	Protocadherin 18
**173**	PGC	Progastricsin (pepsinogen C)
**174**	PHEX	Phosphate regulating endopeptidase homolog, X-linked
**175**	PLA2G5	Phospholipase A2, group V
**176**	PLCB1	Phospholipase C, beta 1 (phosphoinositide-specific)
**177**	PLCE1-AS2	PLCE1 antisense RNA 2]
**178**	PLEKHA1	Pleckstrin homology domain containing, family A (phosphoinositide binding specific) member 1
**179**	PLG	Plasminogen
**180**	PLG	Plasminogen
**181**	PLGLB1	Plasminogen-like B1
**182**	PLGLB1	Plasminogen-like B1
**183**	PPAPDC3	Phosphatidic acid phosphatase type 2 domain containing 3
**184**	PPIF	clone DNA142995 PPIF
**185**	PPP2R2B	Protein phosphatase 2, regulatory subunit B, beta
**186**	PRO2852	Uncharacterized protein PRO2852
**187**	PROKR2	Prokineticin receptor 2
**188**	PRR22	Proline rich 22
**189**	PSORS1C2	Psoriasis susceptibility 1 candidate 2
**190**	PTGS1	Prostaglandin-endoperoxide synthase 1 (prostaglandin G/H synthase and cyclooxygenase)
**191**	PYGM	Phosphorylase, glycogen, muscle
**192**	RALGPS1	Ral GEF with PH domain and SH3 binding motif 1
**193**	RAPGEF2	Rap guanine nucleotide exchange factor (GEF) 2
**194**	RASIP1	Ras interacting protein 1
**195**	RBAK	RB-associated KRAB zinc finger
**196**	RBPMS-AS1	RBPMS antisense RNA 1
**197**	RGAG1	Retrotransposon gag domain containing 1
**198**	RNPC3	RNA-binding region (RNP1, RRM) containing 3
**199**	RORA-AS1	RORA antisense RNA 1
**200**	RSPO1	R-spondin 1
**201**	S1PR1	Sphingosine-1-phosphate receptor 1
**202**	SCG5	Secretogranin V (7B2 protein)
**203**	SCN4A	Sodium channel, voltage gated, type IV alpha subunit
**204**	SELP	Selectin P (granule membrane protein 140kDa, antigen CD62)
**205**	SEPP1	Selenoprotein P, plasma, 1
**206**	SERPINB3	Serpin peptidase inhibitor, clade B (ovalbumin), member 3
**207**	SERPINB4	Serpin peptidase inhibitor, clade B (ovalbumin), member 4
**208**	SIDT1	SID1 transmembrane family, member 1
**209**	SIGLEC5	OB binding protein-2 (OB-BP2)
**210**	SIM1	Single-minded family bHLH transcription factor 1
**211**	SKI	v-ski avian sarcoma viral oncogene homolog
**212**	SLC16A12	Solute carrier family 16, member 12
**213**	SLC16A2	Solute carrier family 16, member 2
**214**	SLC16A4	Solute carrier family 16, member 4
**215**	SLC16A6	Solute carrier family 16, member 6
**216**	SLC47A1	Solute carrier family 47 (multidrug and toxin extrusion), member 1
**217**	SLC6A13	Solute carrier family 6 (neurotransmitter transporter), member 13
**218**	SLC7A7	Solute carrier family 7 (amino acid transporter light chain, y+L system), member 7
**219**	SLC9A8	Solute carrier family 9, subfamily A (NHE8, cation proton antiporter 8), member 8
**220**	SLC9B1	Solute carrier family 9, subfamily B (NHA1, cation proton antiporter 1), member 1
**221**	SLITRK5	SLIT and NTRK-like family, member 5

**222**	SMKR1	Small lysine-rich protein 1
**223**	SOD2	Superoxide dismutase 2, mitochondrial
**224**	SPATA5L1	cDNA FLJ12286 fis, clone MAMMA1001768, weakly similar to CELL DIVISION CYCLE PROTEIN 48 HOMOLOG MJ1156
**225**	SPDYE1	Speedy/RINGO cell cycle regulator family member E1
**226**	SPDYE5	Speedy/RINGO cell cycle regulator family member E5
**227**	SRCIN1	SRC kinase signaling inhibitor 1
**228**	SSH2	Slingshot protein phosphatase 2
**229**	STC2	Stanniocalcin 2
**230**	STX11	Syntaxin 11 (STX11), mRNA [NM_003764]
**231**	TAAR8	Trace amine associated receptor 8
**232**	TAL2	T-cell acute lymphocytic leukemia 2
**233**	TBX19	T-box 19
**234**	TBX3	T-box 3
**235**	TCEAL7	Transcription elongation factor A (SII)-like 7
**236**	TEX28	Testis expressed 28
**237**	THAP5	THAP domain containing 5 (
**238**	TMED3	Transmembrane emp24 protein transport domain containing 3
**239**	TMED6	Transmembrane emp24 protein transport domain containing 6
**240**	TMEM114	Transmembrane protein 114
**241**	TMEM198B	Transmembrane protein 198B, pseudogene
**242**	TMEM30B	Transmembrane protein 30B
**243**	TMEM31	Transmembrane protein 31
**244**	TNFRSF25	Tumor necrosis factor receptor superfamily, member 25
**245**	TNFSF15	Tumor necrosis factor (ligand) superfamily, member 15
**246**	TOB1-AS1	TOB1 antisense RNA 1
**247**	TP53TG3	TP53 target 3
**248**	TPBG	Trophoblast glycoprotein
**249**	TRAF3IP2	TRAF3 interacting protein 2
**250**	TREML3P	Triggering receptor expressed on myeloid cells-like 3
**251**	TRIM10	Tripartite motif containing 10
**252**	TRIM29	Tripartite motif containing 29
**253**	TTC9	Tetratricopeptide repeat domain 9
**254**	TUBAL3	Tubulin, alpha-like 3
**255**	UTS2B	Urotensin 2B
**256**	VNN1	Vanin 1
**257**	WDR49	WD repeat domain 49
**258**	XLOC_l2_013383	BROAD Institute lincRNA
**259**	ZCCHC5	Zinc finger, CCHC domain containing 5
**260**	ZNF484	Zinc finger protein 484
**261**	ZNF563	Zinc finger protein 563
**262**	ZNF586	Zinc finger protein 586
**263**	ZNF596	Zinc finger protein 596
**264**	ZNF605	Zinc finger protein 605
**265**	ZNF738	Zinc finger protein 738

	27-gene signature of MCF7 and MDA-MB-231 BC cells proton treated with 0,5 Gy	
	**GeneSymbol**	**Description**
**1**	COX18	COX18 cytochrome c oxidase assembly factor
**2**	ABCA10	ATP-binding cassette, sub-family A
**3**	ANKRD26	Ankyrin repeat domain 26
**4**	ARHGEF7	Rho guanine nucleotide exchange factor (GEF) 7
**5**	ATL2	Atlastin GTPase 2
**6**	C1orf189	Chromosome 1 open reading frame 189
**7**	C8orf34	Chromosome 8 open reading frame 34
**8**	C9orf131	Chromosome 9 open reading frame 131
**9**	CEP85L	Centrosomal protein 85kDa-like
**10**	COLQ	Collagen-like tail subunit (single strand of homotrimer) of asymmetric acetylcholinesterase
**11**	DDX10	DEAD (Asp-Glu-Ala-Asp) box polypeptide 10
**12**	DNAJC10	DnaJ (Hsp40) homolog, subfamily C, member 10
**13**	DOPEY1	Dopey family member 1
**14**	EN1	Engrailed homeobox 1
**15**	FAM13A-AS1	FAM13A antisense RNA 1
**16**	FLJ36777	Uncharacterized LOC730971
**17**	INPP5D	Inositol polyphosphate-5-phosphatase, 145kDa
**18**	lnc-AC092031,1-1	LNCipedia lincRNA
**19**	lnc-RNF39-4	Non-protein coding RNA 171
**20**	MAGEL2	MAGE-like 2
**21**	MGA	mRNA; cDNA DKFZp667M0810
**22**	MGC16142	Uncharacterized protein MGC16142
**23**	SLC6A13	Solute carrier family 6 (neurotransmitter transporter), member 13
**24**	SSBP2	Single-stranded DNA binding protein 2
**25**	SUV39H2	Suppressor of variegation 3-9 homolog 2
**26**	ZNF546	Zinc finger protein 546
**27**	ZNF563	Zinc finger protein 563

	51-gene signature of MCF7 and MDA-MB-231 BC cells proton treated with 2Gy	
	**GeneSymbol**	**Description**
**1**	ABCA10	ATP-binding cassette, sub-family A
**2**	ACTA1	Actin, alpha 1, skeletal muscle
**3**	ADAMTSL4	cDNA FLJ13544 fis
**4**	AMER2	APC membrane recruitment protein 2
**5**	ARHGEF7	Rho guanine nucleotide exchange factor (GEF) 7
**6**	ATPAF1	ATP synthase mitochondrial F1 complex assembly factor 1
**7**	C1orf86	cDNA FLJ36608 fis, clone TRACH2015824
**8**	C8orf34	Chromosome 8 open reading frame 34
**9**	C9orf131	Chromosome 9 open reading frame 131
**10**	COL20A1	Collagen, type XX, alpha 1
**11**	CTSS	Cathepsin S
**12**	DEFB123	Defensin, beta 123
**13**	DOPEY1	Dopey family member 1
**14**	DUOX2	Dual oxidase 2
**15**	EN1	Engrailed homeobox 1
**16**	FAM13A-AS1	FAM13A antisense RNA 1
**17**	FAM19A2	Family with sequence similarity 19 (chemokine (C-C motif)-like), member A2
**18**	FAM223A	Family with sequence similarity 223, member A (non-protein coding)
**19**	FBLL1	Fibrillarin-like 1
**20**	FIGF	c-fos induced growth factor (vascular endothelial growth factor D)
**21**	FLJ44715	cDNA FLJ44715 fis
**22**	GPR87	G protein-coupled receptor 87
**23**	GPX7	Gutathione peroxidase 7
**24**	HAND1	Heart and neural crest derivatives expressed 1
**25**	HIPK4	Homeodomain interacting protein kinase 4
**26**	INPP5D	Inositol polyphosphate-5-phosphatase, 145kDa
**27**	IRF4	Interferon regulatory factor 4
**28**	LINC00086	Long intergenic non-protein coding RNA 86 (LINC00086), long non-coding RNA
**29**	LINC00421	Long intergenic non-protein coding RNA 421 (LINC00421), long non-coding RNA
**30**	LINC00525	Long intergenic non-protein coding RNA 525 (LINC00525), long non-coding RNA
**31**	lnc-GGCT-1	ALU4_HUMAN (P39191) Alu subfamily SB2 sequence contamination warning entry, partial (40%)
**32**	LOC100128644	Clone DNA147258 LMNE6487 (UNQ6487)
**33**	LOC100506538	Uncharacterized LOC100506538
**34**	LOC344887	NmrA-like family domain containing 1 pseudogene
**35**	LRRC39	Leucine rich repeat containing 39
**36**	LRRC48	Leucine rich repeat containing 48
**37**	MARCH1	Membrane-associated ring finger (C3HC4) 1, E3 ubiquitin protein ligase
**38**	MGC16142	Uncharacterized protein MGC16142
**39**	PGC	Progastricsin (pepsinogen C)
**40**	RNF19B	Ring finger protein 19B
**41**	SCN1B	Sodium channel, voltage gated, type I beta subunit
**42**	SCN4A	Sodium channel, voltage gated, type IV alpha subunit
**43**	SERPINC1	Serpin peptidase inhibitor, clade C (antithrombin), member 1
**44**	SLC6A13	Solute carrier family 6 (neurotransmitter transporter), member 13
**45**	SPIRE1	Spire-type actin nucleation factor 1
**46**	SPTBN5	Spectrin, beta, non-erythrocytic 5
**47**	SSBP2	Single-stranded DNA binding protein 2
**48**	TNFSF15	Tumor necrosis factor (ligand) superfamily, member 15
**49**	TRIM22	Tripartite motif containing 22
**50**	ZNF516	Zinc finger protein 516
**51**	ZNF563	Zinc finger protein 563

	70-gene signature of MCF7 and MDA-MB-231 BC cells proton treated with 9Gy	
	**GeneSymbol**	**Description**
**1**	ABCA10	ATP-binding cassette, sub-family A
**2**	ACR	Acrosin
**3**	ACTA1	Actin, alpha 1, skeletal muscle
**4**	ACY3	Aspartoacylase (aminocyclase) 3
**5**	ADAMTSL4	cDNA FLJ13544 fis
**6**	AMELX	Amelogenin, X-linked
**7**	AMER2	APC membrane recruitment protein 2
**8**	ANKRD26	Ankyrin repeat domain 26
**9**	ANKRD29	Ankyrin repeat domain 29
**10**	ARHGAP30	Rho GTPase activating protein 30
**11**	C12orf5	Chromosome 12 open reading frame 5
**12**	C8orf34	Chromosome 8 open reading frame 34
**13**	C9orf131	Chromosome 9 open reading frame 131
**14**	C9orf41	Chromosome 9 open reading frame 41
**15**	CAPN8	Calpain 8
**16**	CASQ1	Calsequestrin 1 (fast-twitch, skeletal muscle)
**17**	CD22	cDNA: FLJ22814 fis
**18**	CLSTN3	Calsyntenin 3
**19**	CNTN5	Contactin 5
**20**	COL20A1	Collagen, type XX, alpha 1
**21**	COLQ	Collagen-like tail subunit (single strand of homotrimer) of asymmetric acetylcholinesterase
**22**	CTSS	Cathepsin S
**23**	DCLK1	Doublecortin-like kinase 1
**24**	DEFB123	Defensin, beta 123
**25**	DOCK3	Dedicator of cytokinesis 3
**26**	DUOX2	Dual oxidase 2
**27**	EBI3	Epstein-Barr virus induced 3
**28**	EN1	Engrailed homeobox 1
**29**	FAM13A-AS1	FAM13A antisense RNA 1
**30**	FAM223A	Family with sequence similarity 223, member A
**31**	FBLL1	Fibrillarin-like 1
**32**	FGFBP1	Fibroblast growth factor binding protein 1
**33**	FRMD8P1	FERM domain containing 8 pseudogene 1
**34**	GNAO1	Guanine nucleotide binding protein (G protein), alpha activating activity polypeptide O
**35**	GOLGA8A	Golgin A8 family, member A
**36**	GPR52	G protein-coupled receptor 52
**37**	HAND1	Heart and neural crest derivatives expressed 1
**38**	HIPK4	Homeodomain interacting protein kinase 4
**39**	HTR3E	5-hydroxytryptamine (serotonin) receptor 3E, ionotropic
**40**	INIP	Chromosome 9 open reading frame 80
**41**	INPP5D	Inositol polyphosphate-5-phosphatase
**42**	IQCH	IQ motif containing H
**43**	ITGAM	Integrin, alpha M (complement component 3 receptor 3 subunit)
**44**	KCNMA1	Potassium channel, calcium activated large conductance subfamily M alpha, member 1
**45**	LINC00266-1	Long intergenic non-protein coding RNA 266-1
**46**	LINC00421	Long intergenic non-protein coding RNA 421
**47**	lnc-RNF39-4	Non-protein coding RNA 171
**48**	LOC100506538	Uncharacterized LOC100506538
**49**	LOC344887	NmrA-like family domain containing 1 pseudogene
**50**	LOC344887	NmrA-like family domain containing 1 pseudogene
**51**	LOC401317	cDNA clone
**52**	MGC16142	Uncharacterized protein MGC16142
**53**	MMRN2	Multimerin 2

**54**	NCOR1	Nuclear receptor corepressor 1
**55**	OR10C1	Olfactory receptor, family 10, subfamily C, member 1
**56**	PGC	Progastricsin (pepsinogen C)
**57**	RGAG1	Retrotransposon gag domain containing 1
**58**	SCN4A	Sodium channel, voltage gated, type IV alpha subunit
**59**	SLC25A5-AS1	SLC25A5 antisense RNA 1
**60**	SLC6A13	Solute carrier family 6 (neurotransmitter transporter), member 13
**61**	SPIRE1	Spire-type actin nucleation factor 1
**62**	SPRR2G	Small proline-rich protein 2G
**63**	SSH2	Slingshot protein phosphatase 2
**64**	TESPA1	Thymocyte expressed, positive selection associated 1
**65**	TMED3	Transmembrane emp24 protein transport domain containing 3
**66**	TNFRSF13C	Tumor necrosis factor receptor superfamily, member 13C
**67**	TNFSF15	Tumor necrosis factor (ligand) superfamily, member 15
**68**	TPTE2P6	Transmembrane phosphoinositide 3-phosphatase and tensin homolog 2 pseudogene 6
**69**	TRIM22	Tripartite motif containing 22
**70**	VWCE	von Willebrand factor C and EGF domains
