# Supplementary material for: Proton-irradiated breast cells: molecular points of view
Source: J Radiat Res. 2019 May 28;60(4):451–65. doi: 10.1093/jrr/rrz032 (PMC6640903; doi:10.1093/jrr/rrz032)

MCF7 Breast Cancer cell line

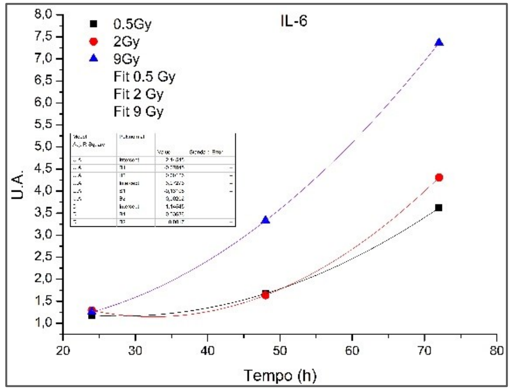

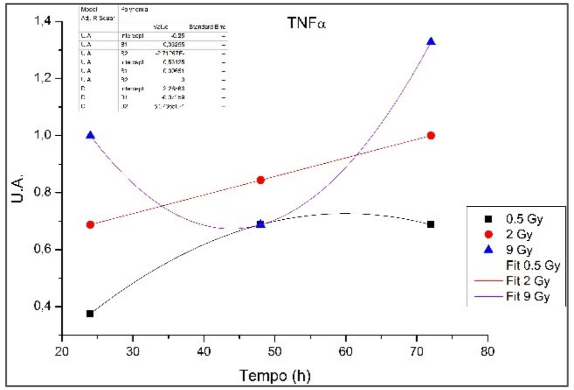

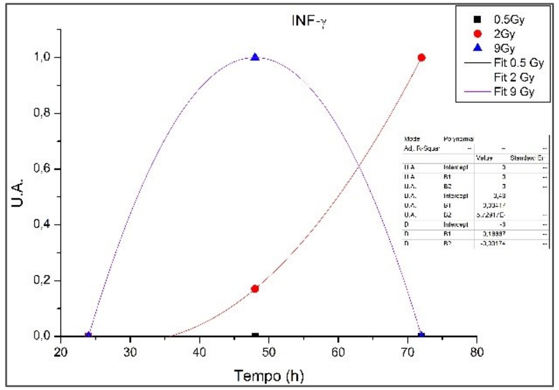

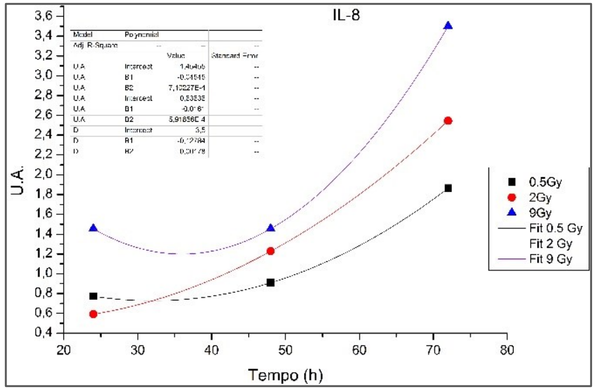

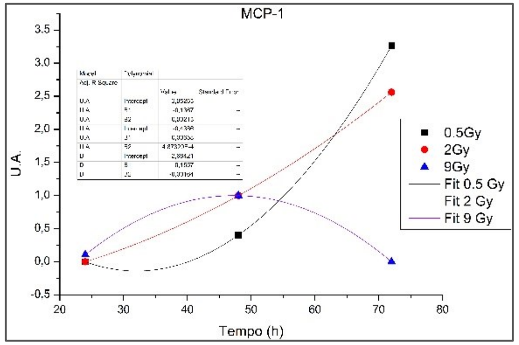

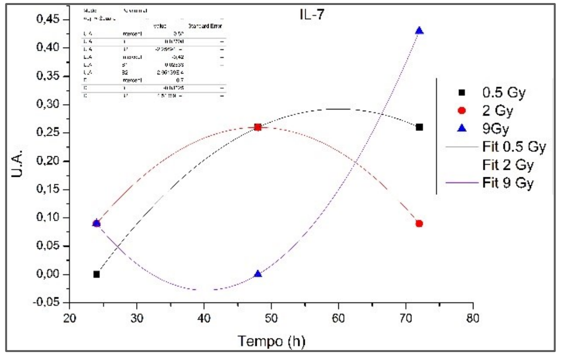

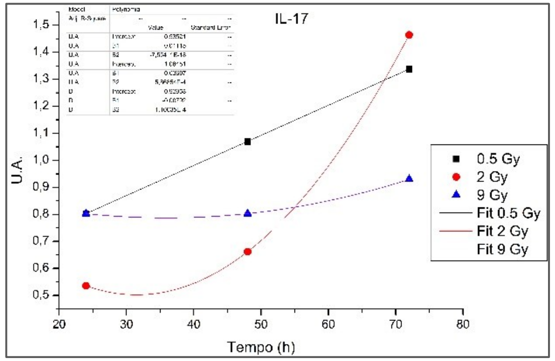

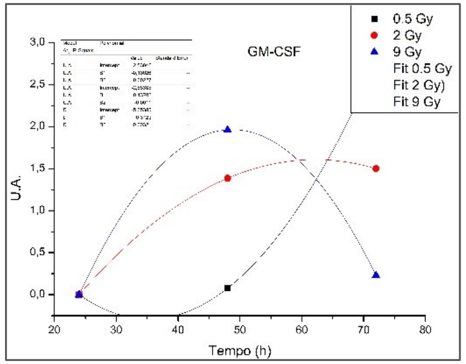

MCF10 A non tumorigenic mammary epithelial cells_I°

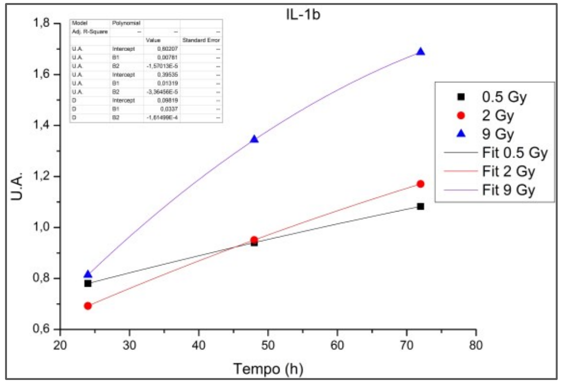

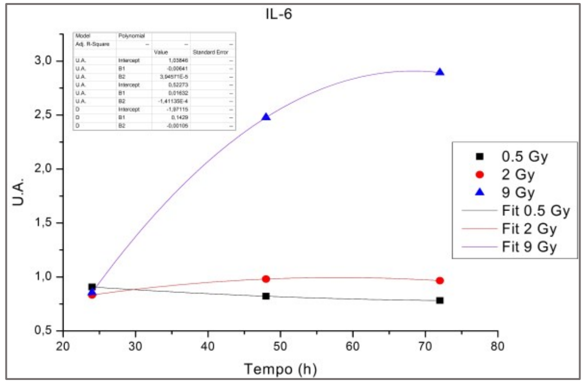

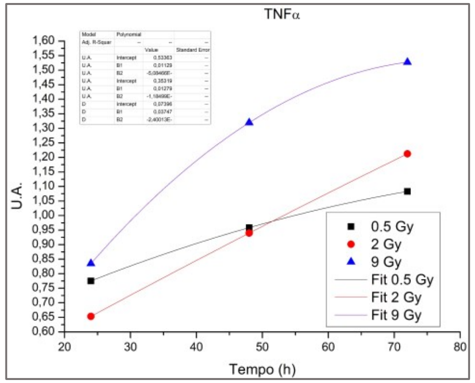

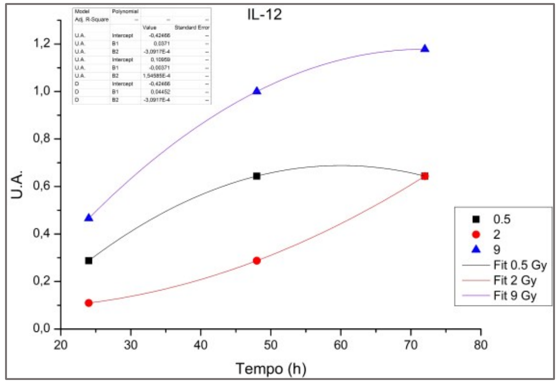

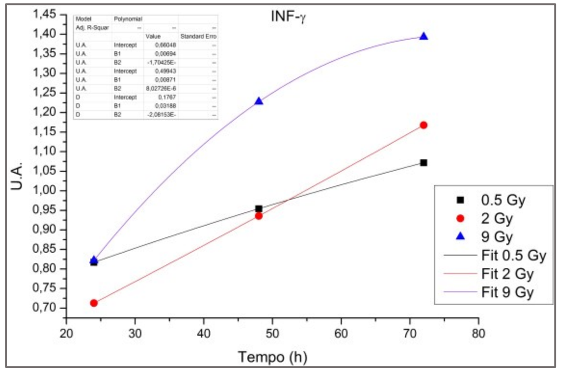

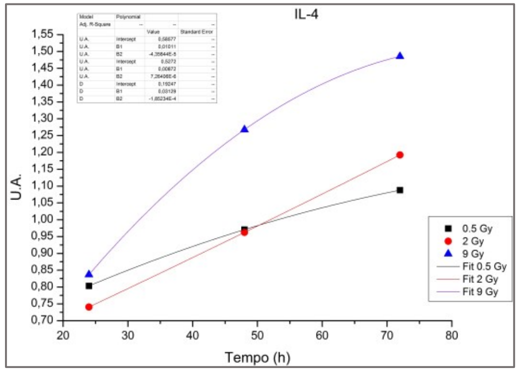

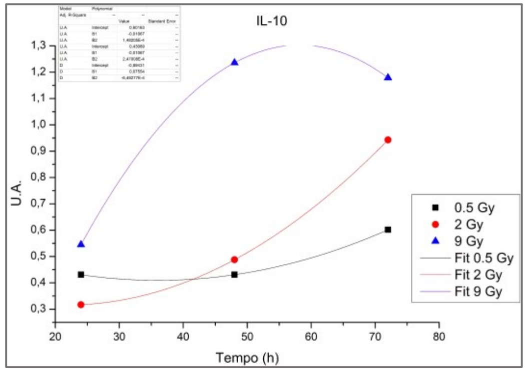

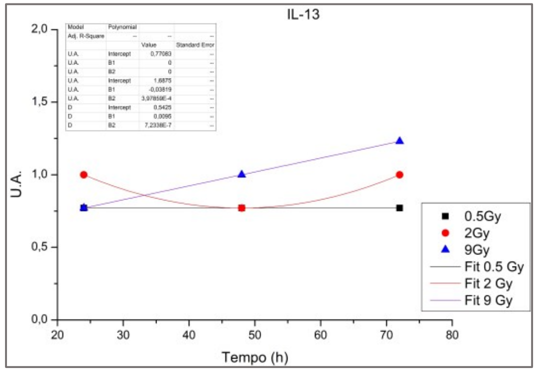

MCF10 A non tumorigenic mammary epithelial cells_II°

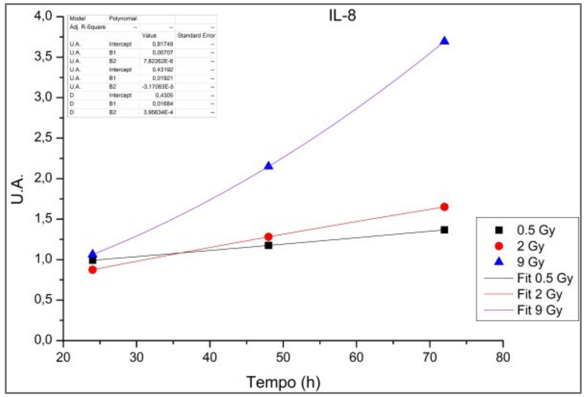

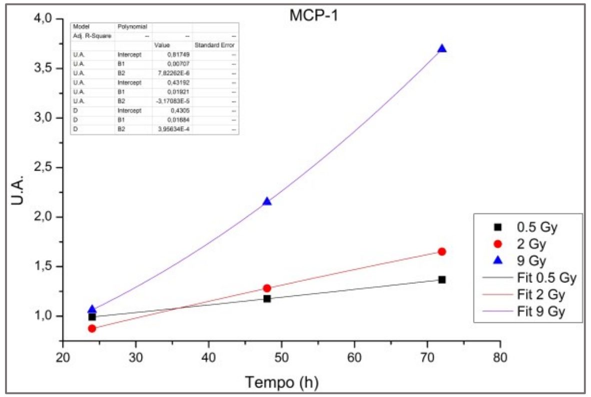

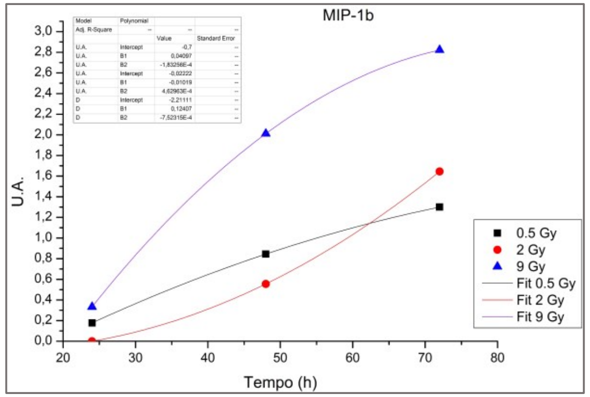

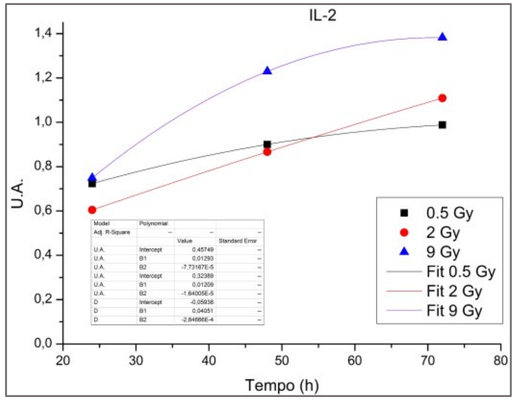

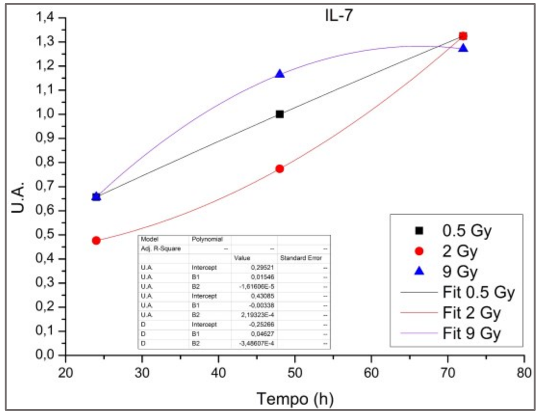

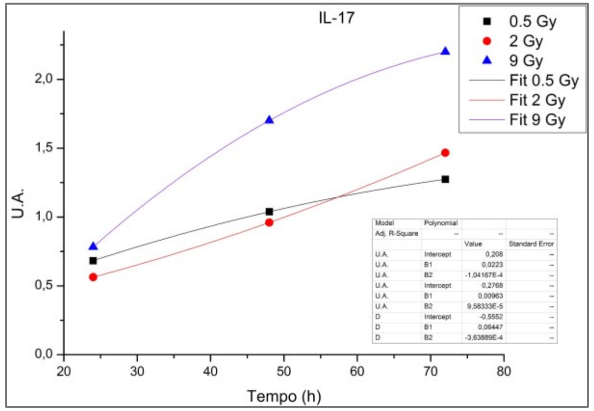

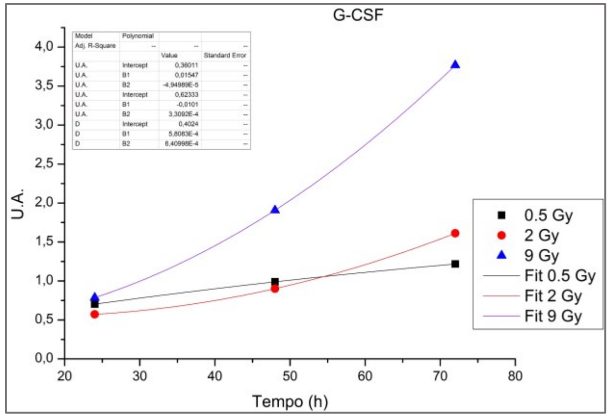

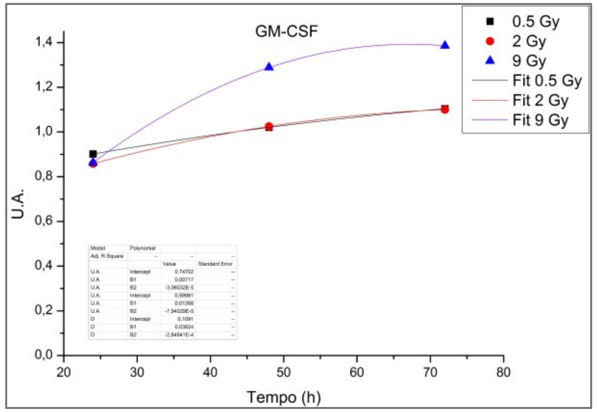

MDA-MB-231 tumorigenic cell line

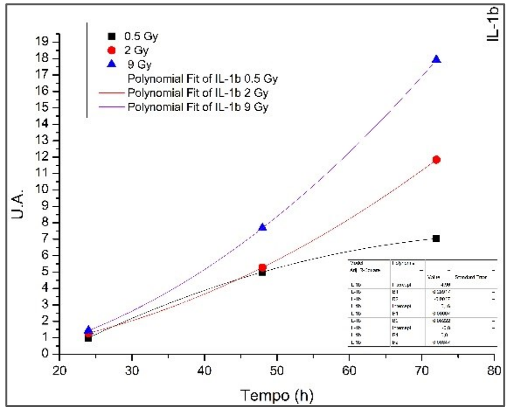

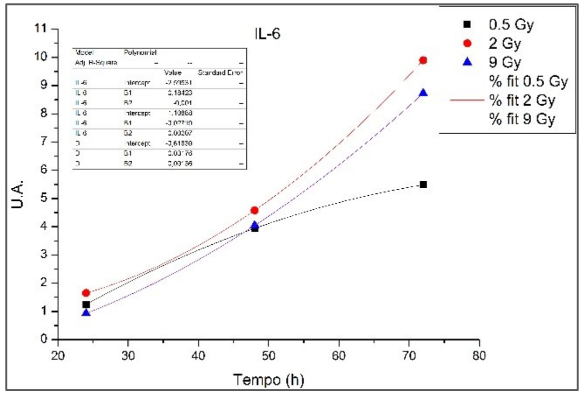

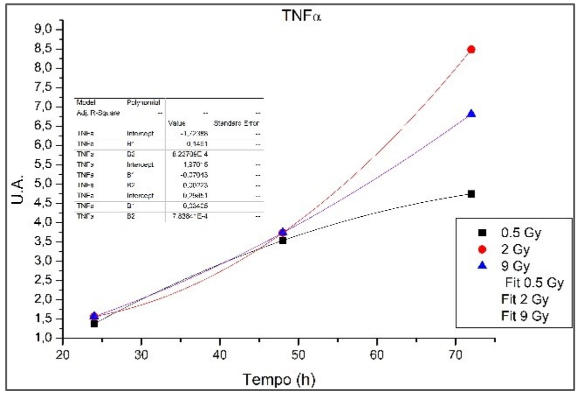

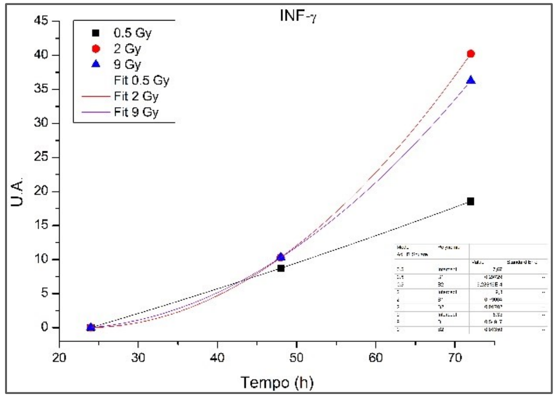

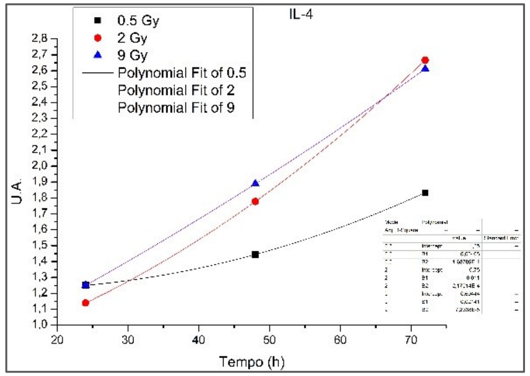

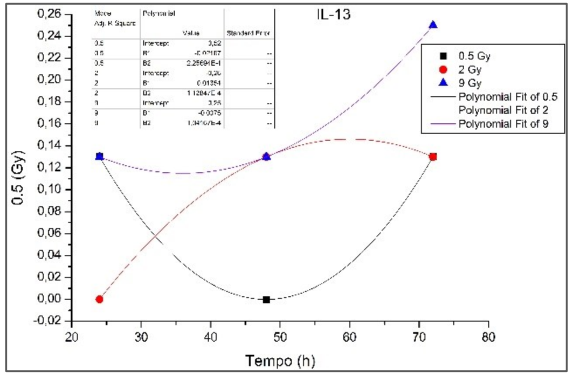

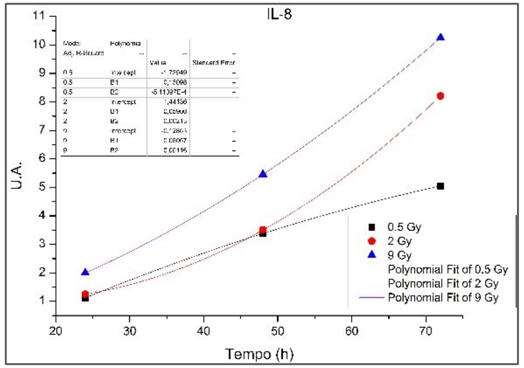

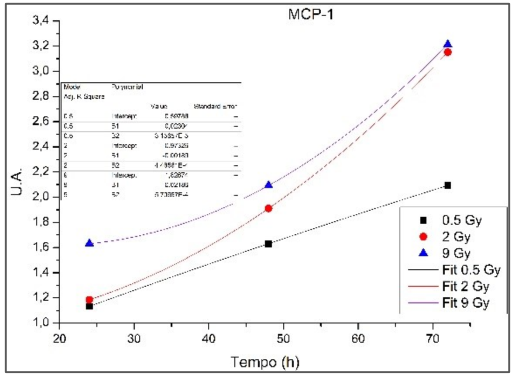

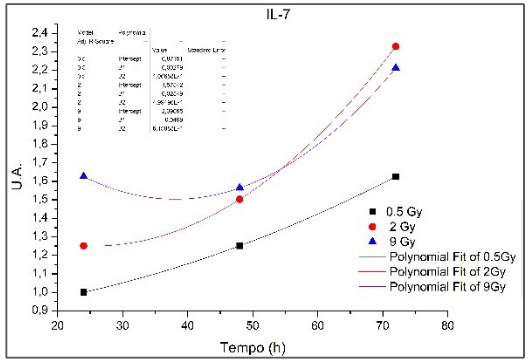

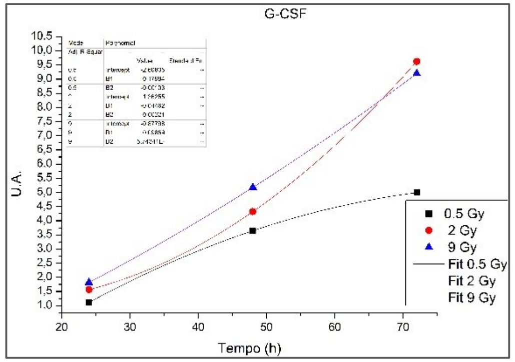

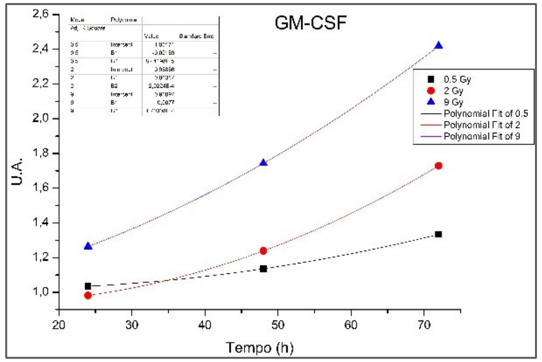

Supplement: Supplementary Data [file rrz032_additional_file_2.pdf]
