# Supplementary material for: Proton-irradiated breast cells: molecular points of view
Source: J Radiat Res. 2019 May 28;60(4):451–65. doi: 10.1093/jrr/rrz032 (PMC6640903; doi:10.1093/jrr/rrz032)
Supplement: Supplementary Data [file rrz032_additional_file_3.pdf]

MCF7 ∩ MDA-MB-231 0,5 Gy treated: 27 gene signature

PubMatrix	Ionizing radiation	Radiation	Cancer	Breast cancer	Proton	Inflammation	Cell cycle	Apoptosis
COX18	0	0	1	0	1	1	1	1
ABCA10	0	1	5	0	0	0	0	0
ANKRD26	0	1	35	1	0	2	1	2
ARHGEF7	0	1	40	8	1	1	44	4
ATL2	0	1	4	1	0	0	2	1
C1orf189	0	0	0	0	0	0	0	0
C8orf34	0	0	1	0	0	0	0	0
C9orf131	0	0	0	0	0	0	0	0
CEP85L	0	0	7	1	0	0	1	0
COLQ	0	0	17	0	0	0	0	2
DDX10	0	0	21	1	0	0	1	1
DNAJC10	0	0	4	0	0	0	1	2
DOPEY1	0	0	1	1	0	0	0	1
EN1	0	3	59	9	0	9	12	20
FAM13A-AS1	0	0	0	0	0	0	0	0
FLJ36777	0	0	0	0	0	0	0	0
INPP5D	0	3	78	10	0	61	6	22
lnc-AC092031	5	29	217	19	3	48	64	66
lnc-RNF39-4	0	0	0	0	0	0	0	0
MAGEL2	0	0	6	1	0	0	4	3
MGA	1	16	140	46	4	16	26	14
MGC16142	0	0	0	0	0	0	0	0
SLC6A13	0	2	1	0	4	1	0	0
SSBP2	1	4	30	2	0	0	3	2
SUV39H2	1	4	39	7	0	3	9	10
ZNF546	0	0	0	0	0	0	0	0
ZNF563	0	0	1	0	0	0	0	0
