# Supplementary material for: Proton-irradiated breast cells: molecular points of view
Source: J Radiat Res. 2019 May 28;60(4):451–65. doi: 10.1093/jrr/rrz032 (PMC6640903; doi:10.1093/jrr/rrz032)
Supplement: Supplementary Data [file rrz032_additional_file_4.pdf]

MCF7 ∩ MDA-MB-231 2 Gy treated: 51 gene signature								
PubMatrix	Ionizing radiation	Radiation	Cancer	Breast cancer	Proton	Inflammation	Cell cycle	Apoptosis
ABCA10	0	1	5	0	0	0	0	0
ACTA1	0	5	10	2	1	10	7	6
ADAMTSL4	0	0	5	0	0	1	0	1
AMER2	0	0	4	0	0	0	0	0
ARHGEF7	0	1	40	8	1	1	44	4
ATPAF1	0	0	2	0	7	0	0	1
C1orf86	0	0	0	0	0	0	0	0
C8orf34	0	0	1	0	0	0	0	0
C9orf131	0	0	0	0	0	0	0	0
COL20A1	0	0	1	1	0	0	0	0
CTSS	2	8	37	6	0	31	5	22
DEFB123	0	0	0	0	0	1	0	0
DOPEY1	0	0	1	1	0	0	0	1
DUOX2	6	18	98	9	9	92	14	48
EN1	0	3	59	9	0	9	12	20
FAM13A-AS1	0	0	0	0	0	0	0	0
FAM19A2	0	0	0	0	0	0	0	0
FAM223A	0	0	0	0	0	0	0	0
FBLL1	0	0	0	0	0	0	0	0
FIGF	0	2	17	7	0	5	7	3
FLJ44715	0	0	0	0	0	0	0	0
GPR87	0	2	17	2	0	1	2	6
GPX7	1	2	20	2	0	5	7	11
HAND1	1	3	36	0	0	3	10	16
HIPK4	0	0	2	0	0	0	0	2
INPP5D	0	3	78	10	0	61	6	22
IRF4	4	20	483	9	2	137	59	96
LINC00086	0	0	3	0	0	0	0	0
LINC00421	0	0	0	0	0	0	0	0
LINC00525	0	0	0	0	0	0	0	0
lnc-GGCT-1	0	0	0	0	0	0	0	0
LOC100128644	0	0	0	0	0	0	0	0
LOC100506538	0	0	0	0	0	0	0	0
LOC344887	0	0	4	1	0	0	1	0
LRRC39	0	0	0	0	0	0	0	0
LRRC48	0	0	0	0	0	0	0	0
MARCH1	0	1	11	0	0	6	1	4
MGC16142	0	0	0	0	0	0	0	0
PGC	14	68	791	77	47	398	286	539
RNF19B	0	0	1	0	0	1	0	1
SCN1B	0	2	9	3	0	1	0	2
SCN4A	0	2	9	1	7	1	2	1
SERPINC1	0	2	15	1	0	6	0	2
SLC6A13	0	2	1	0	4	1	0	0
SPIRE1	0	0	2	0	0	1	2	1
SPTBN5	0	0	2	0	0	0	0	0
SSBP2	1	4	30	2	0	0	3	2
TNFSF15	0	3	78	6	0	89	13	43
TRIM22	1	1	20	2	0	7	9	6
ZNF516	0	0	6	1	0	0	1	0
ZNF563	0	0	1	0	0	0	0	0
