# Supplementary material for: Proton-irradiated breast cells: molecular points of view
Source: J Radiat Res. 2019 May 28;60(4):451–65. doi: 10.1093/jrr/rrz032 (PMC6640903; doi:10.1093/jrr/rrz032)
Supplement: Supplementary Data [file rrz032_additional_file_5.pdf]

MCF7 ∩ MDA-MB-231 9 Gy treated: 70 gene signature								
PubMatrix	Ionizing radiation	Radiation	Cancer	Breast cancer	Proton	Inflammation	Cell cycle	Apoptosis
ABCA10	0	1	5	0	0	0	0	0
ACR	44	470	1189	347	45	491	67	124
ACTA1	0	5	10	2	1	10	7	6
ACY3	0	0	2	0	0	0	0	0
ADAMTSL4	0	0	5	0	0	1	0	1
AMELX	2	17	19	0	4	1	6	2
AMER2	0	0	4	0	0	0	0	0
ANKRD26	0	1	35	1	0	2	1	2
ANKRD29	0	0	0	0	0	0	0	0
ARHGAP30	0	0	5	0	0	1	1	1
C12orf5	4	5	66	9	0	3	12	62
C8orf34	0	0	1	0	0	0	0	0
C9orf131	0	0	0	0	0	0	0	0
C9orf41	0	0	0	0	1	0	0	0
CAPN8	0	1	3	0	0	0	0	1
CASQ1	0	1	6	0	0	3	1	2
CD22	2	51	1205	21	0	72	111	121
CLSTN3	0	0	1	0	0	0	0	2
CNTN5	0	0	2	0	0	2	1	0
COL20A1	0	0	1	1	0	0	0	0
COLQ	0	0	17	0	0	0	0	2
CTSS	2	8	37	6	0	31	5	22
DCLK1	2	11	153	9	0	18	18	29
DEFB123	0	0	0	0	0	1	0	0
DOCK3	0	2	15	0	0	0	2	7
DUOX2	6	18	98	9	9	92	14	48
EBI3	0	2	58	4	0	95	8	10
EN1	0	3	59	9	0	9	12	20
FAM13A-AS1	0	0	0	0	0	0	0	0
FAM223A	0	0	0	0	0	0	0	0
FBLL1	0	0	0	0	0	0	0	0
FGFBP1	1	1	52	7	0	2	8	8
FRMD8P1	0	0	0	0	0	0	0	0
GNAO1	0	0	18	4	0	5	4	4
GOLGA8A	0	0	0	0	0	0	0	0
GPR52	0	0	2	1	0	0	0	0
HAND1	1	3	36	0	0	3	10	16
HIPK4	0	0	2	0	0	0	0	2
HTR3E	0	0	5	1	0	0	0	0
INIP	1	1	2	0	0	0	1	0
INPP5D	0	3	78	10	0	61	6	22
IQCH	0	0	1	0	0	0	0	0
ITGAM	0	14	123	11	4	185	22	53
KCNMA1	2	8	57	8	6	14	13	16
LINC00266-1	0	0	0	0	0	0	0	0
LINC00421	0	0	0	0	0	0	0	0
lnc-RNF39-4	0	0	0	0	0	0	0	0
LOC100506538	0	0	0	0	0	0	0	0
LOC344887	0	0	4	1	0	0	1	0
LOC344887	0	0	4	1	0	0	1	0
LOC401317	0	0	1	0	0	1	1	1
MGC16142	0	0	0	0	0	0	0	0
MMRN2	0	1	11	1	0	1	0	0
NCOR1	3	7	223	62	0	24	59	31
OR10C1	0	0	0	0	0	0	0	0

**PGC**	14	68	791	77	47	398	286	539
**RGAG1**	0	0	1	0	0	0	0	0
**SCN4A**	0	2	9	1	7	1	2	1
**SLC25A5-AS1**	0	0	2	0	0	0	1	1
**SLC6A13**	0	2	1	0	4	1	0	0
**SPIRE1**	0	0	2	0	0	1	2	1
**SPRR2G**	0	0	2	0	0	0	0	0
**SSH2**	0	0	6	0	0	0	1	1
**TESPA1**	0	0	1	0	1	1	0	0
**TMED3**	0	0	4	0	0	0	1	0
**TNFRSF13C**	0	2	71	1	0	32	15	52
**TNFSF15**	0	3	78	6	0	89	13	43
**TPTE2P6**	0	0	0	0	0	0	1	0
**TRIM22**	1	1	20	2	0	7	9	6
**VWCE**	1	2	1	0	0	0	0	0
