# Supplementary material for: Proton-irradiated breast cells: molecular points of view
Source: J Radiat Res. 2019 May 28;60(4):451–65. doi: 10.1093/jrr/rrz032 (PMC6640903; doi:10.1093/jrr/rrz032)
Supplement: Supplementary Data [file rrz032_additional_file_6.pdf]

8 gene signature								
PubMatrix	Ionizing radiation	Radiation	Cancer	Breast cancer	Proton	Inflammation	Cell cycle	Apoptosis
**ABCA10**	0	1	5	0	0	0	0	0
**C8orf34**	0	0	1	0	0	0	0	0
**C9orf131**	0	0	0	0	0	0	0	0
**EN1**	0	3	59	9	0	9	12	20
**FAM13A-AS1**	0	0	0	0	0	0	0	0
**INPP5D**	0	3	78	10	0	61	6	22
**MGC16142**	0	0	0	0	0	0	0	0
**SLC6A13**	0	2	1	0	4	1	0	0
